# Comprehensive analysis of long noncoding RNA expression in dorsal root ganglion reveals cell-type specificity and dysregulation after nerve injury

**DOI:** 10.1097/j.pain.0000000000001416

**Published:** 2018-10-16

**Authors:** Georgios Baskozos, John M. Dawes, Jean S. Austin, Ana Antunes-Martins, Lucy McDermott, Alex J. Clark, Teodora Trendafilova, Jon G. Lees, Stephen B. McMahon, Jeffrey S. Mogil, Christine Orengo, David L. Bennett

**Affiliations:** a Nuffield Department of Clinical Neurosciences, John Radcliffe Hospital, University of Oxford, Oxford, United Kingdom; Departments of b Psychology and; c Anesthesia, Alan Edwards Centre for Research on Pain, McGill University, Montreal, QC, Canada; d Neurorestoration Group, Wolfson Centre for Age-Related Diseases, Institute of Psychiatry, Psychology & Neuroscience, King's College London, London, United Kingdom; e Structural and Molecular Biology, Division of Biosciences, University College London, London, United Kingdom

**Keywords:** LncRNAs, DRG, Animal models of pain, Peripheral neuropathy, SNT, SNI, Transcriptional profiling, Gene expression, IPSC-derived sensory neurons

## Abstract

Dorsal root ganglion (DRG) neurons provide connectivity between peripheral tissues and the spinal cord. Transcriptional plasticity within DRG sensory neurons after peripheral nerve injury contributes to nerve repair but also leads to maladaptive plasticity, including the development of neuropathic pain. This study presents tissue and neuron-specific expression profiling of both known and novel long noncoding RNAs (LncRNAs) in the rodent DRG after nerve injury. We have identified a large number of novel LncRNAs expressed within the rodent DRG, a minority of which were syntenically conserved between the mouse, rat, and human, and including, both intergenic and antisense LncRNAs. We have also identified neuron type–specific LncRNAs in the mouse DRG and LncRNAs that are expressed in human IPS cell–derived sensory neurons. We show significant plasticity in LncRNA expression after nerve injury, which in mice is strain and gender dependent. This resource is publicly available and will aid future studies of DRG neuron identity and the transcriptional landscape in both the naive and injured DRG. We present our work regarding novel antisense and intergenic LncRNAs as an online searchable database, accessible from PainNetworks (http://www.painnetworks.org/). We have also integrated all annotated gene expression data in PainNetworks, so they can be examined in the context of their protein interactions.

## Abstract 2

Supplemental Digital Content is Available in the Text.

Novel and annotated LncRNAs showed marked transcriptional change during neuron differentiation and after nerve injury. Identified LncRNAs were antisense or adjacent to pain genes and ion channels.

## 1. Introduction

Dorsal root ganglion (DRG) neurons provide connectivity between peripheral targets and the spinal cord. These neurons show significant heterogeneity in relation to their morphology, functional properties, growth factor dependence, and transcriptional profile.^1,4,63^ This reflects the highly specialised nature of these neurons subserving distinct sensory modalities, including temperature, pain, itch, touch, and proprioception. Recent single-cell RNA-seq studies have provided a means to classify these neurons and have identified multiple DRG neuron subgroups.^38,67^ Pathologies of DRG neurons—for instance, in the form of acquired or inherited peripheral neuropathies—have a significant impact on human health as a consequence of sensory loss and neuropathic pain^12^ and are destined to become more common with an ageing population and increased prevalence of type II diabetes.

A wide variety of injuries applied to sensory neurons, whether traumatic or metabolic, result in marked alterations in transcription of protein-coding genes.^13,34,53^ Such changes can have either beneficial or maladaptive outcomes, including increased expression of regeneration-associated genes^9,51^ and altered expression of ion channels resulting in enhanced DRG neuronal excitability and neuropathic pain. The DRG has therefore become a model system to study the transcriptional changes after injury. This focus on RNAs' encoding proteins is understandable, given their obvious link with function. However, there are other types of RNA and one of these, long noncoding RNA (LncRNA) has been relatively neglected and little studied in the context of sensory neurones.

LncRNAs are usually multiexonic transcripts of more than 200 base pairs that can modulate gene expression through *cis* and *trans* signalling, and have important functional effects.^6,31,58,69,76^ The mechanisms by which LncRNAs may alter gene expression are very diverse, including: complementary binding of antisense LncRNAs, transcriptional interference at promoter sites, altered chromatin structure, competing for miRNA binding, and binding to transcription factors.^26,44,81^

Ion channels are key determinants of the excitability and hence the functional properties of sensory neurons. Antisense LncRNAs have previously been identified to the voltage-gated potassium channel, KCNA2,^86^ and the voltage-gated sodium channel, SCN9A.^32^ In the former case, induction of the antisense LncRNA after nerve injury was shown to result in reduced expression of KCNA2 (which acts as an excitability break), leading to sensory neuronal hyperexcitability and the development of neuropathic pain. These are selected examples of functionally relevant LncRNAs that illustrate the important role they can play. However, there has, to date, been no comprehensive analysis of LncRNA expression within the DRG partly because LncRNAs are typically expressed at low levels and are known to vary by species, tissue, and developmental stage.^31,68^ Our aim was to use high-coverage RNA-seq combined with a dedicated bioinformatics platform to identify as comprehensively as possible LncRNAs expressed in the DRG. We compared rats and 2 different mouse strains (which show differing degrees of mechanical hypersensitivity after nerve injury^67^). We wished to determine whether LncRNAs were expressed in a cell-type–specific manner and also to assess the effect of nerve injury on LncRNA expression. In addition, we also assessed LncRNA expression in human Induced Pluripotent Stem Cell –derived sensory neurons.

## 2. Material and methods

Seventeen supplementary spreadsheets are available at http://doi.org/10.6084/m9.figshare.6508205, and 18 tables, 10 figures, and supplementary methods are in supplementary digital content (available at http://links.lww.com/PAIN/A676).

### 2.1. Data availability

Data are in GSE107182 super series that consists of GSE107180 (rodents' DRG) and GSE107181 (human IPSC). Splicing junctions (SJ), differentially expressed (DE) data analysis results, and GTF files with annotations used are included as downloadable supplementary files of the GEO series GSE107180 and GSE107181. Supplemental data spreadsheets are available at http://doi.org/10.6084/m9.figshare.6508205.

IGV genome browser tracks for novel LncRNAs are publicly available.

Mouse: https://storage.googleapis.com/lncrnatracks/Mouse_DRG_LncRNAs/Novel_lncRNAs_mouse_DRG.gtf (from IGV version 2.4x); gs://lncrnatracks/Mouse_DRG_LncRNAs/Novel_lncRNAs_mouse_DRG.gtf (until IGV version 2.3x).

Rat: https://storage.googleapis.com/lncrnatracks/Rat_DRG_LncRNAs/Novel_lncRNAs_rat_DRG.gtf (from IGV version 2.4x); gs://lncrnatracks/Rat_DRG_LncRNAs/Novel_lncRNAs_rat_DRG.gtf (until IGV version 2.3x).

Human IPSC: https://storage.googleapis.com/lncrnatracks/Human_IPS_LncRNAs/Novel_lncRNAs_IPS.gtf (from IGV version 2.4x); gs://lncrnatracks/Human_IPS_LncRNAs/Novel_lncRNAs_IPS.gtf (until IGV version 2.3x).

Processed RNA-seq mouse DRG samples for IGV genome browser are publicly available at: https://storage.googleapis.com/lncrnatracks/Mouse_DRG_BAM/Sample50_BALB.c_SHAM_M.sorted.bam; https://storage.googleapis.com/lncrnatracks/Mouse_DRG_BAM/Sample55_BALB.c_SHAM_F.sorted.bam; https://storage.googleapis.com/lncrnatracks/Mouse_DRG_BAM/Sample58_B10.D2_SHAM_M.sorted.bam; https://storage.googleapis.com/lncrnatracks/Mouse_DRG_BAM/Sample77_B10.D2_SHAM_F.sorted.bam; https://storage.googleapis.com/lncrnatracks/Mouse_DRG_BAM/Sample65_BALB.c_SNI_F.sorted.bam; https://storage.googleapis.com/lncrnatracks/Mouse_DRG_BAM/Sample70_BALB.c_SNI_M.sorted.bam; https://storage.googleapis.com/lncrnatracks/Mouse_DRG_BAM/Sample78_B10.D2_SNI_F.sorted.bam; and https://storage.googleapis.com/lncrnatracks/Mouse_DRG_BAM/Sample90_B10.D2_SNI_M.sorted.bam.

All data have been integrated to PainNetworks. In http://www.painnetworks.org, the user can examine a gene (or set of genes) of interest alongside known interaction partners on the protein level. This information is displayed by the resource in the form of a network. Moreover, the user can access all expression data (log2 fold change and false discovery rate–adjusted *P* values) and download these in the form of spreadsheets. A tutorial on how to use PainNetworks can be accessed following this link http://www.painnetworks.org/tutorials/RefMan.pdf.

All intergenic and antisense LncRNAs' profiling data are accessible in PainNetworks (http://www.painnetworks.org) → ExpressionData → Mouse centric/Rat centric/Human centric. Experiment names are GB-BALBC-LNCRNAS for the BALB/c mouse, GB-SNI-B10D2-LNCRNAS for the B10.D2 mouse, GB-RAT-LNCRNAS for the rat, and IPSC_HS_AD2-LNCRNAS for IPSC-derived neurons. Naming is as follows: Closest {gene or sense gene}_LNCRNA_{IG or nothing}_chr:start-end(strand). Examples:

ENSMUSG00000000093_LNCRNA_IG:11:85830666-85831495(+) is the intergenic LncRNA with coordinates 11:85830666-85831495(+) close to the ENSMUSG00000000093 gene.

ENSMUSG00000000094_LNCRNA:11:85897018-85900613(−) is the antisense LncRNA with coordinates 11:85897018-85900613(−) on the opposite strand of ENSMUSG00000000094 gene.

### 2.2. Animals: welfare, tissue, and sample collection

#### 2.2.1. Rat

All procedures on rats were performed in accordance with UK home office regulations and in line with the Animals Scientific Procedures Act 1986 at a licensed facility at King's College London. Animals were group housed in temperature- and humidity-controlled rooms where food and water was available ad libitum, with a 12-hour light–dark cycle. The welfare of all animals was continually assessed throughout all procedures. In total, 24 rats were used.

Rats were humanely culled. L5 DRG tissue from male Wistar rats was collected 21 days after the spinal nerve transection (SNT) surgery, placed into sterile tubes, frozen on dry ice, and stored at −80°C. Each sample comprises 3 pooled animals, and we had 4 samples of each condition (SNT vs sham).

#### 2.2.2. Mouse

All procedures in mice were performed in McGill University, Montreal, Canada, were approved by the McGill University Animal Care Committee and are fully consistent with Canadian Council on Animal Care guidelines. All mice strains were procured from Jackson Laboratories (Bar Harbor, ME) at 4 to 8 weeks of age. All animals of the same sex were group housed in a vivarium at ≈21°C in standard shoebox cages, 2 to 4 per cage, with access to food (Harlan Teklad 8604; Envigo, Huntington, United Kingdom) and tap water ad libitum. Average weights were 20.2 (SD = 2.89) for BALB/c mice and 21.4 (SD = 3.48) for B10.D2 mice (N = 12 per strain; for each strain, 6 spared nerve injury [SNI]–6 Sham stratified for sex). All operations were performed on adult mice. Brain and DRG tissue has also been dissected from 3 wild-type mice (C57/bl6) and was used to determine relative expression of mRNA using quantitative real-time polymerase chain reaction (qPCR) (see below). In total, 27 mice were used.

The tip of the iliac bone, “the first articular process more than 1 mm rostral to the iliac crest,”^61^ was used as the landmark for identifying the L5 DRG in all samples. L3 and L4 DRG were dissected from all mice 28 days after peripheral nerve injury. Each sample represents 1 animal and consists of both L3 and L4 DRG. Twelve BALB/c mice and 12 B10.D2 mice stratified for condition and sex were used. All dissections were performed on dry ice, and RNase Decontamination Solution was used to prevent RNA degradation. Tissue was placed into sterile Eppendorf tubes and initially stored on dry ice. For long-term storage, samples were stored in a −80°C freezer.

#### 2.2.3. IPS-derived human neurons

Human fibroblast–derived IPSC was generated as described previously.^11^ Neural differentiation was performed using^8^ the protocol with modifications.

### 2.3. Animal models of pain

#### 2.3.1. Mouse spared nerve injury

The surgical procedure for SNI followed a published protocol developed for rats^15^ and adapted for mice.^64^ Under general anaesthesia (isoflurane and oxygen), the common peroneal and the sural branch of the sciatic nerve were cut and the tibial branch spared. For sham surgery, the same surgical and anaesthetization procedures were followed, but the nerve branches were simply exposed and not damaged. We assessed mechanical hypersensitivity after SNI surgery on the ipsilateral mouse paw.

#### 2.3.2. Rat spinal nerve transection

The left L5 spinal nerve was ligated and transected, and the L4 and L6 branches were left intact. In sham animals, the spinal nerve was exposed but not ligated.

#### 2.3.3. Behavioural tests

The behavioural test was conducted in a specially allocated room in the animal facility unit at McGill University, performed at a consistent time of the day and by the same experimenter. Mice habituated to the vivarium for at least 1 week before testing. Mechanical pain–related hypersensitivity in mice was assessed using von Frey filaments and the up-down method of Dixon^10^ to determine the 50% withdrawal threshold. Mice were first acclimatised to behaviour equipment and baseline behaviour performed 3 times and an average was calculated before surgery. Baseline paw withdrawal threshold was 1.27 g (SD = 0.22) for BALB/c strain and 1.36 g (SD = 0.23) for B10.D2 strain (N = 12). Mice were assigned to the sham or SNI group randomly and postinjury mechanical sensitivity tested at day 1, 7, 14, 21, and 28 (N = 12 per strain stratified for sex and condition, 6 SNI–6 Sham mice per strain). Assuming an effect of 30% and an SD = 20%, we need an N = 6 to achieve power = 80 at an a = 0.05 two-sided 1-way analysis of variance (ANOVA). Mice from both strains and surgery groups were tested on the same day. The experimenter was not informed about the condition (injury vs sham) of animals but could not be blinded because of the coat colour of the different strains.

### 2.4. RNA isolation and library preparation

RNA was extracted using a hybrid method of phenol extraction (TriPure; Roche, Welwyn Garden City, United Kingdom) and combined with column purification (High Pure RNA tissue Kit; Roche).^14^ Dorsal root ganglion samples were first homogenised in TriPure using a handheld homogeniser (Cole-Palmer, Saint Neots, United Kingdom). For IPS cells, TriPure was added directly to the well after removal of media. The concentration of RNA in the samples was measured using a nanodrop. Total RNA was provided to the sequencing centre, and the ribodepleted fraction was selected for further sequencing. In rats, this was the polyadenylated fraction. It was then converted to cDNA using the strand-specific deoxy-UTP strand-marking protocol.

### 2.5. Sequencing and mapping

All samples were sequenced at the Oxford Genomics Centre. Sequencing was performed using the Illumina HiSeq4000 paired-end protocol with 100 bp reads for the mouse DRG, 75 bp for human IPSC/neurons, and Illumina HiSeq2000–100 bp reads for the rat DRG.

The DRG from 24 mice (12 per strain stratified for sex and condition) was sent for sequencing. During library preparation, 2 samples (sample 72 BALB/c SNI Male and sample 68 B10.D2 SNI Female) were accidentally mixed together and destroyed. From the 22 samples sent for sequencing, 2 were excluded (sample 59 B10.D2 SHAM Male and sample 66 BALB/c SNI Female) because of having more ambiguously mapped reads, lower percentage of mapped reads, and higher Cook's distance than all the other samples.

Mapping to the genome was done using STAR aligner.^18^ Reads were mapped on the mm10 mouse genome, rn6 rat genome, and Hg38 human genome, all downloaded from ENSEMBL. Conditions and strains were multiplexed in lanes and library batches. Lanes were merged as BAM files after mapping.^39^

### 2.6. Differentially expressed and counting features

Differentially expressed analysis was performed using DESeq2^42^ default settings. Significant cutoff in all cases was false discovery rate–adjusted *P* value <0.05. Counting of features was done using HTSeq^3^ and the intersection and not empty strategy to resolve ambiguously counted reads.

All visualisations used regularised log2-transformed counts.^42^ Principal component analysis was always performed on regularized log-transformed counts using the top 10,000 genes in mice and humans, and 5000 genes in rats ranked by their SD. Hierarchical clustering was done on regularised log2 counts of the whole gene set, using Euclidean distances and complete linkage.

Gene Ontology (GO) enrichment for DE genes was carried in R using top GO and GSEA.^2,49^ Enrichment of neuron subtype–specific genes was calculated with the Fisher exact test, and enrichment of Biological Process (BP) in network modules was calculated using hypergeometric distribution.

### 2.7. Identification of novel LncRNAs

We used a customised reference–based transcript assembly pipeline that requires a reference genome and gene set annotations. Workflow similar^7,22,29^ with modifications to produce annotations at the gene level. Doing this, we get a nonredundant annotation of unique genes of LncRNAs suitable for count-based DE analysis.^3,42^ The concept of islands of expression (I.o.E) is described in Ref. 22. Coverage cutoff has been set as in Ref. 7.

Only properly paired and uniquely mapped reads were selected. We selected SJs covered with >2 reads and with lengths >20 and <100,000. We discarded all reads overlapping annotated gene models. We then used the remaining subset of RNA-seq reads to identify I.o.E outside known gene models using a coverage window approach. Gene models were extended by 1000 bp in each direction to ensure that elongated untranslated regions (UTRs) or not yet annotated exons would not be considered putative novel genes. We selected continuous regions above the coverage threshold of more than a read-mate length to ensure that overlapping read mates would not artificially increase coverage. For I.o.E length ≥100 and depth >2, I.o.E were identified using the function “BAM_to_IOE.” Islands of expression were collapsed and clustered as co-overlapping features connected by SJs. A connectivity matrix was created holding all interconnecting I.o.E. In each cluster, consensus introns were calculated by the relative frequency of each discrete segment of a set of SJs. We then subtracted the genomic intervals of these consensus introns from the genomic intervals of the grouped (I.o.E) to reconstruct full-length putative LncRNAs. For novel I.o.E with no overlapping SJs, we first selected only the intersect of the respective genomic regions across all samples. Then, monoexonic putative LncRNAs were kept for further analysis only if the length-normalised coverage had Pr (>) < 0.1. Coverage across I.o.E was fed into a smoothed z-score signal processing algorithm. *Z*-score thresholding was used to identify introns not identified by the aligner and sudden coverage drops indicating end of transcription activity.^5^ Rolling coverage was calculated over a smoothing window of 31 bp, and the minimum coverage drop threshold was set to 5 and the minimum intron length to 20 bp. We only kept novel intronic genes if they were supported by evidence of novel splicing junction and did not contain retained introns.

We included putative LncRNAs in this novel annotation only if they were present in all replicates of a biological condition or strain. Annotations were exported in the Gene Transfer Format (GTF). Subsequently, we filtered out transcripts with length <200 bp, and we used CPAT^72^ to assess coding potential. An average expression cutoff threshold similar to Ref. 56 of >0.5 fpkm for at least one condition was applied to novel LncRNAs performed for downstream analysis. The pipeline was scripted in R^59^ using bioconductor^21^ packages and custom scripts. All iterative processes were executed in parallel to optimise run times using parallel and BiocParallel.^48,50,56^ More details in supplementary methods are (available at http://links.lww.com/PAIN/A676). All scripts of the workflow are available in github: http://github.com/gbaskozos/Scripts_LncRNAs.

#### 2.7.1. Transcription start sites mapping to mm10

Transcription start sites (TSS) data were downloaded from FANTOM 5 database.^20,40^ We downloaded TSS data that have been classified as “True TSS” by the “TSS classifier.” The UCSC Lift Over tool^46^ has been used to translate genomic coordinates from the mm9 genome to the mm10. Fifty-one percent of the true TSS were unambiguously mapped to mm10.

### 2.8. Tissue specificity

Tissue specificity was calculated using the tau metric^77^:
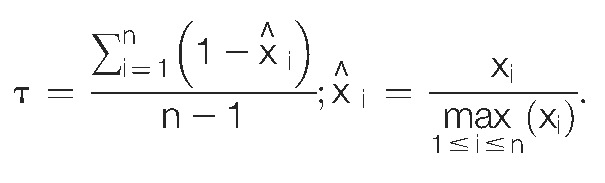


### 2.9. Gene coexpression network

WGCNA^23^ was used to create a weighted gene coexpression network. Analysis was performed only in mice, as it requires an n >15. Weighted bicorrelation was used as a robust correlation metric. An unsigned network was constructed using only genes that had >10 counts in 25% of samples. Then, the top 25% of genes ranked according to their median absolute deviations were used for the analysis. Scale-free topology was achieved with a soft threshold = 5. Modules detected with hierarchical clustering and dynamic tree cut, minimum module size = 30, cut height = 0.995, and deep split = 2. Merged threshold was 0.2. Module eigengenes were calculated as the first principal component of each module. Module membership of LncRNAs was calculated as the absolute (unsigned) bicorrelation with the module eigengenes. The hypergeometric distribution and the Fisher exact test were used to identify the top GO-enriched (*P* value < 0.05) terms for each module.

### 2.10. Primer design

Primers for the detection of LncRNA and reference gene expression were designed using Primer-BLAST (https://www.ncbi.nlm.nih.gov/tools/primer-blast/). Primers were designed not to overlap any other annotated gene. Thus, primers designed for antisense LncRNAs were not able to detect regions complementary to sense gene exons. Primer efficiency and specificity were validated before experimental use.

### 2.11. Reverse transcription polymerase chain reaction

For qPCR analysis, RNA samples were converted into cDNA using Evoscript Universal cDNA Master kit (Roche) and by following the manufacturer's instructions. This kit uses random primers. For LncRNA2754, the primers designed also detected a putative UTR, and therefore, a strand-specific real-time reaction was used. Strand-specific RT primers (see table 18, available at http://links.lww.com/PAIN/A676) were used for LncRNA2754 and for HPRT1 (final concentration 0.5 µM), and 200 ng of RNA was used for each reaction. Strand-specific reverse translation into cDNA synthesis was performed using Transcriptor reverse transcriptase (Roche) and dNTPs (Promega, Southampton, United Kingdom).

### 2.12. Quantitative real-time polymerase chain reaction

For qPCR analysis, cDNA (5 ng) and primer pairs (1 μM, see table 18, available at http://links.lww.com/PAIN/A676) were mixed with LightCycler 480 SYBR Green Master (Roche) in a 1:1 ratio and added to white 384-well plates (Roche). Plates were run on a 45-cycle protocol using the LC 480 II system (Roche). Gene expression for each mouse target primer was normalized against the reference gene HPRT1, and the relative expression (delta CT) was calculated. For human IPS cells, transcript expression was normalised against the average CT of GAPDH and HPRT1. For each target, transcript expression is shown relative to a control group (eg, Sham). Significance was calculated using ANOVA with a design of ∼ sex + condition for each mouse strain and ∼ condition for human IPSC averaging over all cell lines. N = 10 per strain (6 Sham–4 SNI for BALB/c, 5 Sham–5 SNI for B10.D2; for strand-specific qPCR, B10.D2 strain N =8, 5 Sham–3 SNI). Quantitative real-time polymerase chain reaction was also used to assess relative mRNA expression in brain vs DRG, N = 3. Significance was calculated using 1-way ANOVA.

### 2.13. In situ hybridisation

In situ hybridization was performed as previously shown.^14^ Once cut, sections were air-dried onto superfrost slides in the cryostat for 0.5 hours and then stored in the −80°C freezer. In situ hybridization was performed using the RNAScope 2.5 RED chromogenic assay kit and by following the manufacturer's instructions (Advanced Cell Diagnostics, Newark, CA). Briefly, slides containing tissue sections were removed from the −80°C freezer, allowed to equilibrate to RT and rehydrated in phosphate-buffered saline (PBS). Pretreatment required a hydrogen peroxide step at RT, followed by an antigen retrieval step and protease treatment in a hybridization oven at 40°C. Slides were then incubated with the target or control probes at 40°C for 2 hours. For HAGLR mRNA, the probes were designed to target position 1153 to 2443 of NR_110445.1. After probe incubation, slides were subjected to 6 rounds of amplification, and the probe signal was developed via a reaction with fast red. To combine with immunohistochemistry (IHC), tissue sections were then washed with PBS-Tx (0.3%) and treated with either the isolectin B4 (IB4) conjugated to biotin (Sigma, Gillingham, United Kingdom, 1:100) or primary antibodies against NF200 (mouse anti-NF200, Sigma, 1:250) and CGRP (rabbit anti-CGRP; Peninsula Labs, San Carlos, CA, 1:1000) overnight at room temperature. IB4 and primary antibodies were diluted in PBS-Tx (0.3%). Slides were then washed with PBS-Tx (0.3%) and then incubated with the appropriate secondary antibody (anti-mouse Pacific Blue or ant-rabbit Alexa 488; Thermo Fisher Scientific, Abingdon, United Kingdom) or a Pacific blue-streptavidin (Thermo Fisher Scientific) at a concentration of 1:500 for 3 to 4 hours at room temperature. Slides were then washed, mounted using Vectashield, and imaged using a confocal microscope (Zeiss, Cambridge, United Kingdom).

## 3. Results

### 3.1. Novel long noncoding RNAs expressed in the rodent dorsal root ganglion

To identify unknown transcribed regions outside known gene models (ie, representation of RNA transcripts produced by a gene) and then group them into transcriptional units representing putative novel LncRNAs on the gene level, we performed RNA-sequencing (RNA-Seq) of DRG tissue harvested from animal models of nerve injury vs sham controls. We profiled novel LncRNAs alongside known annotated protein-coding genes and LncRNAs from the ENSEMBL genome database.^79^ To obtain computational predictions of novel LncRNAs, we used a reference-based customised pipeline^45^ that relies on a reference genome and gene set annotations. It uses the output of the STAR^18^ aligner, and the quality of predictions is dependent on the aligner accuracy, the quality of the reference genome, and the completeness of the gene set annotation. A coverage threshold is used^7^ to identify nonannotated continuously transcribed regions,^22^ ie, I.o.E., and then clustered together and trimmed these regions using de novo identified SJs from mapping the RNA-seq reads to the organism's genome. We also applied a signal processing, smoothed *z*-score thresholding algorithm to further identify coverage drops (putative introns and transcription ends) and peaks (putative exons). To identify nonannotated I.o.E and to differentiate them from UTRs or not-yet-annotated exons belonging to known gene models, we filtered out reads overlapping ENSEMBL and RefSeq annotations as well as genomic regions of 1000 bp from the 5′ and 3′ end of known gene models. Doing so, we have excluded any predictions overlapping with a region of 1000 bp from both ends of annotated gene models. The mean length of 3′ UTRs is 424 bp and 524 bp in rodents and humans, respectively. For the 5′ UTRs, mean lengths are 127 bp in rodents and 146 bp in humans.^55^ We should note that we used both major annotation consortia, so an incomplete gene annotation (ie, missing exons, UTRs, and isoforms) in just one of the annotated gene sets would not influence results. Given the fact that we discarded all predictions overlapping annotated genes, we could not also identify extracoding RNAs, as these overlap protein-coding genes on the sense strand.^62^

32.6% of read pairs on average were overlapping regions outside gene models (as we used paired-end data, the units of evidence for gene expression are always pairs of reads). This finding is consistent with Ref. 22.

**Figure 1.**
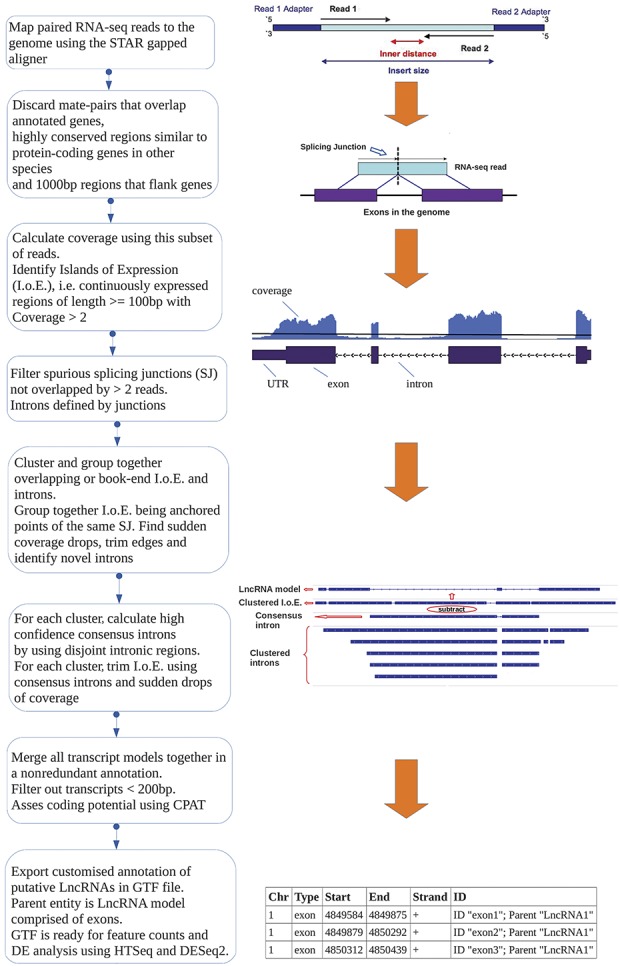
Overview of the computational pipeline used for the identification of novel LncRNAs using RNA-seq data. See Materials and Methods—Identification of novel LncRNAs and Supplementary Methods, available at http://links.lww.com/PAIN/A676, identification of novel LncRNAs. DE, differentially expressed; LncRNAs, long noncoding RNAs; UTR, untranslated region.

To acquire a complete representation of the nonannotated transcribed regions, we intentionally applied a low coverage threshold (>2 sequence coverage for the region) for at least the length of an RNA-seq read (≥100 bp). The cutoff threshold is similar to the one applied in Ref. 22. Then, we clustered and trimmed these regions using splicing information from novel SJs identified by at least 2 RNA-seq read-pairs and a smoothed *z*-score over a rolling window to identify coverage drops. To predict and analyse LncRNAs on the gene level, we merged together all identified transcripts from individual samples to create a unified set of nonredundant, novel, putative LncRNAs in the form of a GTF file. The bioinformatic workflow is illustrated in Figure 1; for more details, see methods.

We then calculated the coding potential and performed transcriptional profiling to identify DE novel and known gene models between animal models of peripheral neuropathy and control (sham surgery) samples. To perform DE analysis without overestimating fold changes for lowly expressed transcripts, we used the analysis software DESeq2^42^ and filtered DE results according to expression levels and consistency. Significant cutoff for DE was adjusted *P* value <0.05 in all cases. We filtered out novel LncRNAs that were not expressed in at least all replicates of a condition or strain or were below an average expression threshold of >0.5 fpkm in at least one condition. We particularly focused on antisense LncRNAs, ie, overlapping exonic regions of the gene on the opposite strand, and intergenic LncRNAs, lying on the intergenic space between known gene models. All expression data including antisense and intergenic LncRNAs are available in http://www.painnetworks.org,^54^ and all RNA-seq data (raw data and the whole gene set, ie, novel LncRNAs and annotated genes) are available in Gene Expression Omnibus (GEO) (GSE107182, GSE107180, and GSE107181). Supplemental spreadsheets of the complete data set are available at http://doi.org/10.6084/m9.figshare.6508205.

LncRNAs are known to have relatively poor conservation between species, so we aimed to compare one rat (Wistar) and 2 mouse strains (BALB/c and B10.D2 strains). RNA-seq quality was good for both experiments (supplementary tables 1–4, available at http://links.lww.com/PAIN/A676). Quality was assessed based on: the percentage of uniquely mapped reads (89% for mice and 72.51% for rats), the number of properly and concordantly paired reads, the average Phred score for read quality (32.2 for mice and 34.3 for rats), the base calling at the extremities of reads (0.08 for mice and 0 for rats, low Phred scores at the end of reads), the median of the insert between paired read mates (192.1 for mice and 153.4 for rats), and the GC content of reads for all sequencing lanes and samples (48.2% for mice and 51.4% for rats). Two mouse samples were excluded because they had much lower mapped reads (sample 59: 73.3%), a very high percentage of unmapped reads because they were too short (sample 59: 18.1%), much more reads mapped to multiple loci (sample 66 and sample 59), and higher Cook's distance (supplementary figure 1, available at http://links.lww.com/PAIN/A676). Uniquely mapped read pairs were used for the downstream analysis. Raw and processed gene expression data are available in GEO.

In total, we had on average 64 million uniquely aligned pairs of reads per sample for mice and 41 million pairs of reads for rats, more than enough both for identification of de novo LncRNAs and then to ask whether they were DE in injury vs sham conditions.^19^

**Figure 2.**
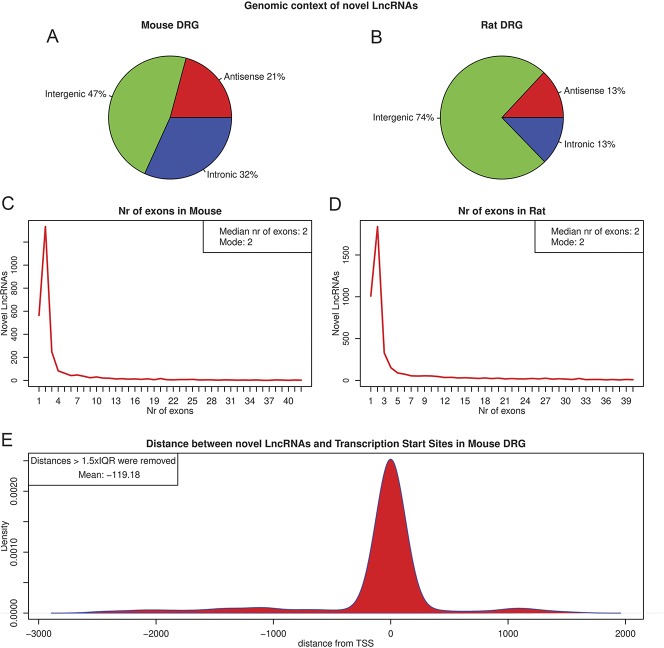
Attributes of novel LncRNAs identified in mice and rats. (A and B) Classification of novel LncRNAs according to genomic context in mice (A) and rats (B). (C and D) Exon distribution of novel LncRNAs in mice (C) and rats (D). (E) Kernel density of distances between novel LncRNAs and 5′ CAGE TSS. Distance is measured in genomic bases. Outlying distances >1.5 × IQR of the distribution were removed (all data in supplementary figure 2 available, at http://links.lww.com/PAIN/A676). Median distance of TSS is 0 bp of the predicted LncRNA transcript. DRG, dorsal root ganglion; IQR, interquartile range; LncRNAs, long noncoding RNAs; TSS, transcription start sites.

Using this approach, we initially identified and reconstructed thousands of nonannotated transcribed loci in the mouse and rat DRG with length of more than 200 bp. Then, we evaluated whether these novel transcripts were protein coding or noncoding. After coding potential calculation using the coding potential calculator CPAT,^72^ we obtained 6657 long consistently expressed transcripts classified as noncoding in the rat DRG and 4729 in the mouse DRG. Four thousand three hundred fifteen of these passed the expression threshold in rats and 2693 in mice and were retained for the downstream analysis. These long novel transcripts with no coding potential were considered putative novel LncRNAs. The majority of novel LncRNAs were intergenic in both species, with 21% and 13% antisense (overlapping exonic regions of protein-coding genes on the opposite strand) in the mouse and rat DRG, respectively (Figs. 2A and B). Most novel LncRNAs were multiexonic, with a distribution of exons heavily skewed towards biexonic transcripts (Figs. 2C and D). This exon distribution is very similar to GENCODE findings.^27^

The usage of ribodepleted RNA for the library preparation allowed us to completely sample and interrogate the noncoding transcriptome and led to a relatively high proportion of intronic noncoding transcripts being identified.^29^

To increase confidence and to gather more evidence regarding the completeness and expression of predicted novel LncRNAs, we examined the relationship between their genomic loci and annotated TSS. To do this, we used 5′ CAGE (cap analysis gene expression) TSS data^40^ that are available in the mouse. CAGE is a technique for high-throughput identification of sequence tags corresponding to 5′ ends of RNA at the cap sites and the identification of the TSS.^65^ As TSS data were not available for the mouse reference genome mm10, we translated and mapped coordinates from the mm9 genome to mm10. We then calculated the kernel density of the distance between the TSS that were mapped to mouse genome mm10 (51% of the TSS available for mm9) and a region within 100 bp of the 5′ end of the putative LncRNA similarly to Ref. 27. For both known and novel LncRNAs, the kernel density was highest at 0 but with more spread for the novel LncRNAs (Fig. 2E, supplementary Figure 2; available at http://links.lww.com/PAIN/A676). 33.7% of the antisense and intergenic LncRNAs had a predicted TSS on their 5′ end on the same strand of the genome. When we removed the outlying distant TSS—ie, more than 1.5 times the interquantile range of the distribution—the mean distance of TSS and novel LncRNAs was 119 bp upstream of the predicted transcript. In a GENCODE study,^27^ 15% of identified LncRNAs in humans had a TSS on their 5′ end. These results highlight how close the 5′ end of novel LncRNAs was to experimentally determined TSS. Due to the fact that only a fraction of TSS were mapped to the current mouse genome, these data are likely to be an underestimate. These findings are in concordance with those of Ref. 17. We also note that a significant fraction of novel LncRNAs are either incomplete, not yet annotated extended UTRs or that LncRNAs are indeed independent transcription units arising from TSS that are not yet annotated, possibly due to their low expression level.

**Figure 3.**
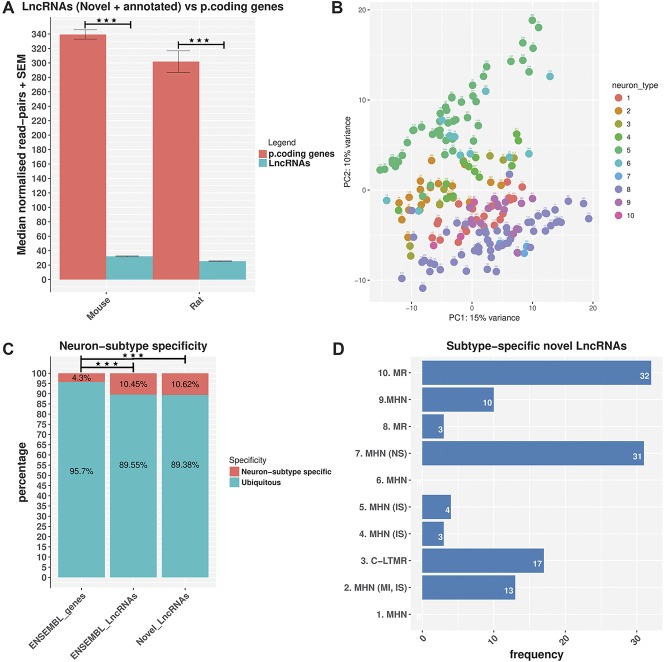
Features of LncRNA's expression. (A) Median read counts of LncRNAs (novel and ENSEMBL annotated) vs protein-coding genes in mice and rats. Data are presented as mean plus SEM. Significance was assessed with the Mann–Whitney *U* test (MWW). (B) Principal component analysis plot of the expression of novel LncRNAs in different DRG neuron subtypes. Neuron subtypes are as follows: 1. MHN, 2. MHN (MI, IS), 3. C-LTMR, 4. MHN (IS), 5. MHN (IS), 6. MHN, 7. MHN (NS), 8. MR, 9. MHN, and 10. MR. Axis represents PCs and shows percentage of original data's variance explained by the respective PC (PCA plot of ENSEMBL genes in supplementary figure 3A, available at http://links.lww.com/PAIN/A676). (C) Neuron subtype specificity (tau > 0.8, average log 2 counts > 3 for at least one neuron subtype). Enrichment was assessed with the Fisher exact test for count data. Kernel density of the Tau specificity metric in supplementary figure 3B (available at http://links.lww.com/PAIN/A676). (D) Distribution of neuron subtype–specific novel LncRNAs in different neuron subtypes. C-LTMR, c-fiber low-threshold mechanoreceptors; DRG, dorsal root ganglion; IS, itch sensitive; LncRNAs, long noncoding RNAs; MHN, mechanoheat receptors; MI, mechanically insensitive; MN, mechanical nociceptor; MR, mechanoreceptor; MS, mechanically sensitive; PCA, principal component analysis. *P* < 0.05*, *P* < 0.01**, and *P* < 0.001***.

In both species, we found that LncRNAs were significantly and consistently expressed at a lower level than protein-coding genes (Fig. 3A). This ratio of about 10-fold lower expression of LncRNAs to protein-coding genes is similar to previous studies.^69,76^

Nociceptors are a major component of the DRG, and a number of pain genes are selectively expressed by these neurons. We therefore studied the genomic context of identified LncRNAs, and in particular, whether they were antisense or in close genomic proximity with known pain genes downloaded from the Pain Genes Database.^35^ The Pain Genes Database catalogues genes that have been shown to have an impact on pain-related behaviour in rodents based on transgenic knockout experiments. Of the 449 genes in the database, we have found 13 novel LncRNAs antisense to pain genes in mice and 19 in rats (supplementary tables 5 and 6, available at http://links.lww.com/PAIN/A676). Twenty-three intergenic LncRNAs had a pain gene as their closest genomic neighbour in mice and 57 in rats (supplementary tables 7 and 8 available at http://links.lww.com/PAIN/A676). Ion channels are key determinants of sensory neuron excitability. We identified LncRNAs antisense to voltage-gated sodium channels, potassium channels, calcium channels, chloride channels, and TRP channels, within the mouse and rat DRG, as shown in supplementary tables 9 and 10 (available at http://links.lww.com/PAIN/A676).

In general, we had modest syntenic conservation (ie, in equivalent genomic positions) between species. We used synteny portal^37^ to retrieve synteny blocks conserved between the human (GRCh38.88), mouse (mm10), and rat (rn5) with a resolution of 150,000 bp. We lifted genomic coordinates from rn5 to rn6 genome and found in total 912 conserved synteny blocks in humans and mice and 443 uniquely mapped in the current rat genome. Eight hundred (18.5%) novel LncRNAs in rats and 1271 (47%) in mice were in these syntenic blocks conserved between the 3 species. Moreover, 649 LncRNAs in mice and 782 in rats were in 200 common syntenic blocks between the 2 species (supplementary data available at http://doi.org/10.6084/m9.figshare.6508205, S. Data 1). Five hundred nine LncRNAs in rats and 397 in mice were antisense of the same orthologous genes in mice and rats (S. Data 2 and 3, available at http://doi.org/10.6084/m9.figshare.6508205).

### 3.2. Different types of dorsal root ganglion neurons selectively express LncRNAs

Dorsal root ganglion neurons are heterogenous both in terms of physiology and molecular profile; to identify whether the expression of LncRNA may be DRG subtype–specific, we reanalysed a published data set of single-cell RNA sequencing data^38^ derived from C57BL/6 mouse DRG neurons for expression of the novel LncRNAs that we had identified. The authors had previously generated 10 different DRG neuron subtypes from their analysis of 197 neurons. We found that we could effectively identify transcriptome- and size-based neuronal types based on the selective expression of both ENSEMBL annotated genes and our novel LncRNAs.

Principal component analyses of the expression set of novel LncRNAs showed that samples belonging to most of the sensory neuron subtypes were clustered together (Fig. 3B) but with a higher spread than for annotated genes (supplementary figure 3A, available at http://links.lww.com/PAIN/A676), suggesting that these can be important transcriptional units the expression of which is highly related to the different subtypes of DRG neurons.

We then used the Tau tissue specificity metric^77^ to identify highly DRG subtype–specific LncRNAs expressed in the mouse DRG. The density plot of the Tau index distribution (supplementary figure 3B, available at http://links.lww.com/PAIN/A676; and S. Data 4, available at http://doi.org/10.6084/m9.figshare.6508205) suggested that the Tau >0.8 was an appropriate threshold for separating neuron subtype–specific from ubiquitous annotated genes and novel LncRNAs. To consider a gene or LncRNA neuron subtype–specific, we also imposed a cutoff mean expression threshold of >3 log2-normalised read pairs across all samples of a neuronal subtype. Eighty-seven novel LncRNAs, 119 annotated LncRNAs, and 597 protein-coding genes were neuron subtype–specific. This reveals a significant (Fisher exact test *P* value <0.001) enrichment of novel and annotated LncRNAs among the neuron subtype–specific transcripts (Fig. 3C). This quantification of the DRG subtype specificity confirms that LncRNAs' expression pattern is more tissue specific than protein-coding genes'. In studying the different DRG subtypes (as defined by Li et al., 2016), we noted that there was an uneven distribution of subtype-specific LncRNAs, Figure 3D.

### 3.3. IPSC-derived sensory neurons differentially express known and novel LncRNAs compared with their respective IPS cells

**Figure 4.**
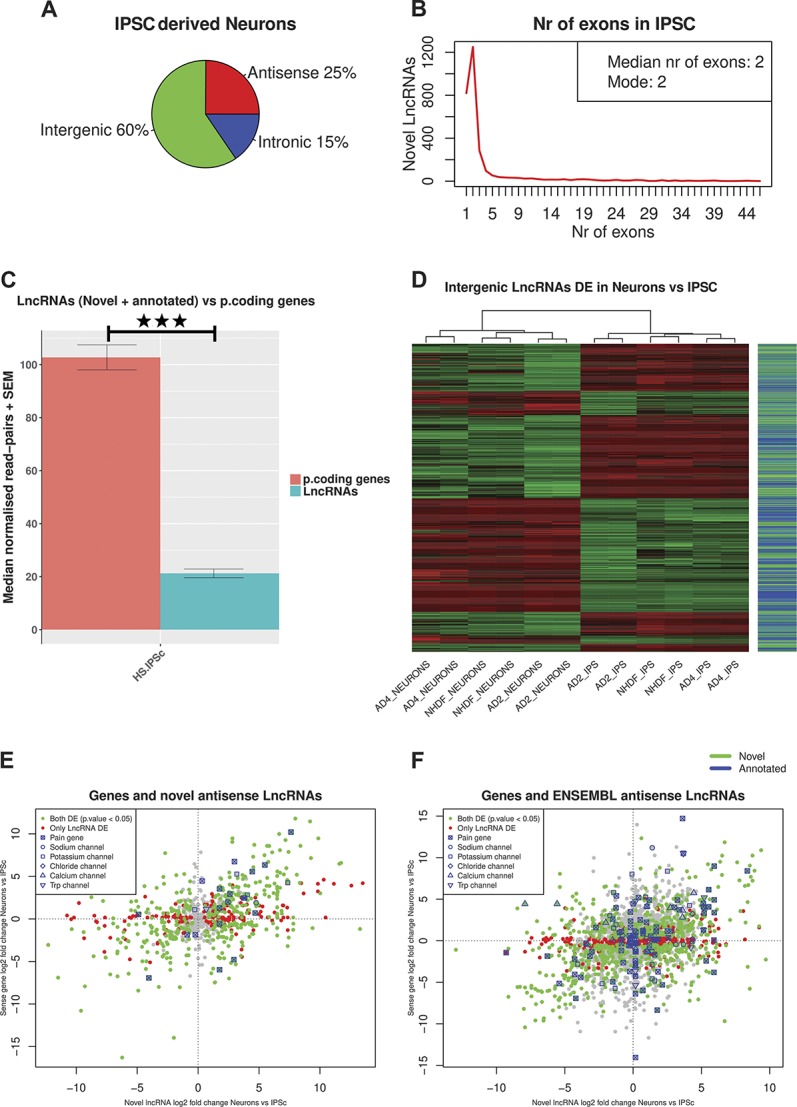
Expression patterns of LncRNAs in human IPSC–derived neurons. (A) Classification of novel LncRNAs according to genomic context. (B) Exon distribution of novel LncRNAs. (C) Median read counts of LncRNAs (novel and ENSEMBL annotated) vs protein-coding genes in human IPSC–derived neurons. Data are presented as mean plus SEM. Significance was assessed with the Mann–Whitney *U* test (MWW). (D) Heatmap of novel and annotated intergenic LncRNAs DE between human IPSC-derived neurons and IPSC. (E and F) Expression plot of novel antisense LncRNAs (E) and annotated ENSEMBL antisense LncRNAs (F) vs the sense protein-coding gene. LncRNAs antisense to DE genes *Kcnj6* and *Trpm3* were DE with opposite log2 fold changes to the DE sense gene. *P* < 0.05*, *P* < 0.01**, and *P* < 0.001***. DE, differentially expressed; LncRNAs, long noncoding RNAs.

We assessed LncRNA expression in human IPSC–derived sensory neurons generated from healthy individuals. We followed a differentiation protocol known to produce neurons with a gene expression profile and functional characteristics that are very similar to rodent sensory neurons.^8,11,80^ Virtually, all the resulting neurons express the sensory neuron marker Brn3a, project extensively arborized neurites, a subset can be myelinated and exhibit mature electrophysiological characteristics of sensory afferents.^8,73^ At least 84% of the ion channel genes known to be expressed in the human DRG were shown to be expressed by sensory neurons generated using this protocol.^80^ We compared gene expression in IPSC-derived sensory neurons with expression in IPSC parent lines to focus on LncRNAs enriched in differentiated sensory neurons (GEO GSE107181, S. data 5–7, available at http://doi.org/10.6084/m9.figshare.6508205). We first interrogated genes annotated in the ENSEMBL GRCh38.88 gene set, and we also identified and profiled 2948 novel LncRNAs in human IPSC and IPSC-derived sensory neurons, most of them were intergenic (Fig. 4A) and biexonic (Fig. 4B). Again, we found that LncRNAs were significantly lower expressed than protein-coding RNAs (Fig. 4C). IPSC-derived sensory neurons from 3 different cell lines (identified as AD2, AD4, and NHDF) were very similar to each other and considerably different to the IPSC parent lines (supplementary figure 4A, available at http://links.lww.com/PAIN/A676). Forty-two percent of all expressed genes were DE in IPSC-derived sensory neurons vs IPSCs, which is a remarkable transcriptional change. A total of 6103 annotated LncRNAs (ENSEMBL GRCh38.88) were consistently expressed in at least all samples of either IPSCs or IPSC-derived sensory neurons. A total of 1830 (30%) of them were significantly DE in all 3 cell lines between IPSC-derived sensory neurons and IPSCs; the majority of these were intergenic (47%). Seventy-seven percent of the expressed novel LncRNAs were significantly DE between IPSC-derived sensory neurons and IPS cells. All 3 cell lines had in common 371 novel LncRNAs significantly DE.

In both annotated and novel intergenic LncRNAs, distance was modestly but significantly anticorrelated with expression correlation with their closest genomic neighbour (supplementary figure 4B, C; available at http://links.lww.com/PAIN/A676). Two thousand seven hundred forty-three intergenic LncRNAs (novel and annotated) were significantly DE between IPSC-derived neurons and IPSC (Fig. 4D). Eighty annotated and 16 novel LncRNAs were antisense to pain genes (S. Data 8, available at http://doi.org/10.6084/m9.figshare.6508205), and 15 annotated and 18 novel LncRNAs had a pain gene as its closest genomic neighbour (S. Data 9, available at http://doi.org/10.6084/m9.figshare.6508205). Eight of the LncRNAs antisense to pain genes were significantly DE between IPSC-derived sensory neurons and IPSCs with a significantly DE gene on the opposite strand and anticorrelated expression changes, 1 of them was novel (supplementary table 11, available at http://links.lww.com/PAIN/A676). IPSC-derived sensory neurons expressed LncRNAs (including both annotated and novel LncRNAs) antisense to TRP, voltage-gated sodium, potassium, chloride, and calcium ion channels; in some cases, these showed the opposite expression changes relative to the sense gene on the opposite strand (Figs. 4E and F).

**Figure 5.**
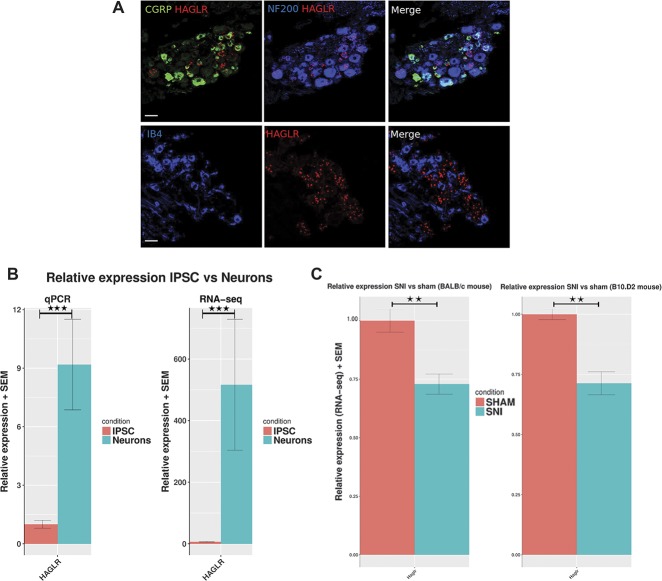
(A) In situ hybridisation for HAGLR LncRNA (mouse ortholog of human HAGLR) shows expression in the mouse WT L4 DRG. The ISH signal was developed using a fast red reaction. From right to left: Representative images of mouse DRG sections stained for HAGLR (red), NF200 (blue), CGRP (green), and IB4 (blue). Scale bar 50 µm. (B) Quantification of expression change of HAGLR in human IPSC vs IPSC-derived neurons. Relative expression assessed by qPCR of HAGLR LncRNA. (C) RNA-seq determined relative expression in SNI vs Sham BALB/c and B10.D2 mice DRG. Data are presented as mean plus SEM. *P* < 0.05*, *P* < 0.01**, and *P* < 0.001***. DRG, dorsal root ganglion; ISH, in situ hybridization; LncRNAs, long noncoding RNAs; qPCR, quantitative real-time polymerase chain reaction; SNI, spared nerve injury.

One example of the antisense LncRNAs, the HOXD Cluster Antisense RNA 1 (HAGLR) was further investigated because it was very highly upregulated in neurons vs IPSC, and HoxD genes have been implicated in nociceptor specification.^25^ HAGLR was ranked first among the significantly DE annotated antisense LncRNAs by its log2 fold change (8.74) and base mean counts (261.5). Differential expression was validated by qPCR (Fig. 5, supplementary table 12, available at http://links.lww.com/PAIN/A676). In situ hybridization also revealed that it was expressed by mouse DRG cells in vivo, and it was found to be significantly downregulated in the mouse DRG after nerve injury (see below as well as Fig. 5).

We also observed similar extent of syntenic conservation between the human, mouse, and rat in novel LncRNAs identified in human IPSC and IPSC-derived sensory neurons. One thousand three hundred twelve (44.5%) were in syntenically conserved blocks between the 3 species. Four hundred fifty-nine LncRNAs in human IPSC and 495 in mice were in 126 common syntenic blocks between the 2 species (S. Data 10, available at http://doi.org/10.6084/m9.figshare.6508205), and 522 were antisense orthologous genes in humans and mice (S. Data 11, available at http://doi.org/10.6084/m9.figshare.6508205).

Human DRG eQTLs (expression quantitative trait loci) have recently been identified and associated with pain phenotypes.^52^ We interrogated this data set for overlaps with novel and annotated LncRNAs expressed in IPSC-derived sensory neurons, as this may provide an underlying mechanism through which a single nucleotide polymorphism (SNP) at this site may impact on gene expression. We only considered overlaps valid if they were found on exons of the respective transcript. We found that 4 annotated LncRNAs (3 antisense and 1 intergenic) that had DRG eQTLS were expressed in IPSC-derived sensory neurons, and also that 5 novel LncRNAs were overlapping DRG eQTLS (Table 1).

**Table 1:**
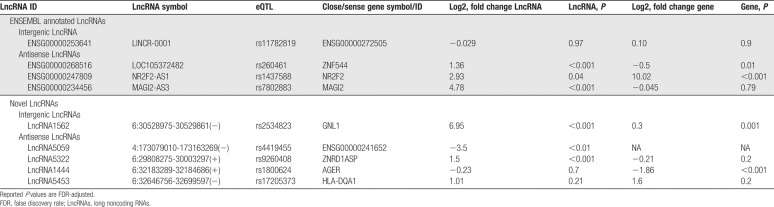
Annotated and novel LncRNAs overlapped by eQTLs.

### 3.4. Expression profiling of LncRNAs after nerve injury

We then assessed differential expression of LncRNAs within the DRG after nerve injury both in rats and mice. We used the SNI model in mice and the SNT model in rats. We used 2 different mouse strains: BALB/c and B10.D2, which have previously been shown to develop different levels of mechanical hypersensitivity after nerve injury.^67^

#### 3.4.1. Transcriptional changes of LncRNAs in the rat dorsal root ganglion

**Figure 6.**
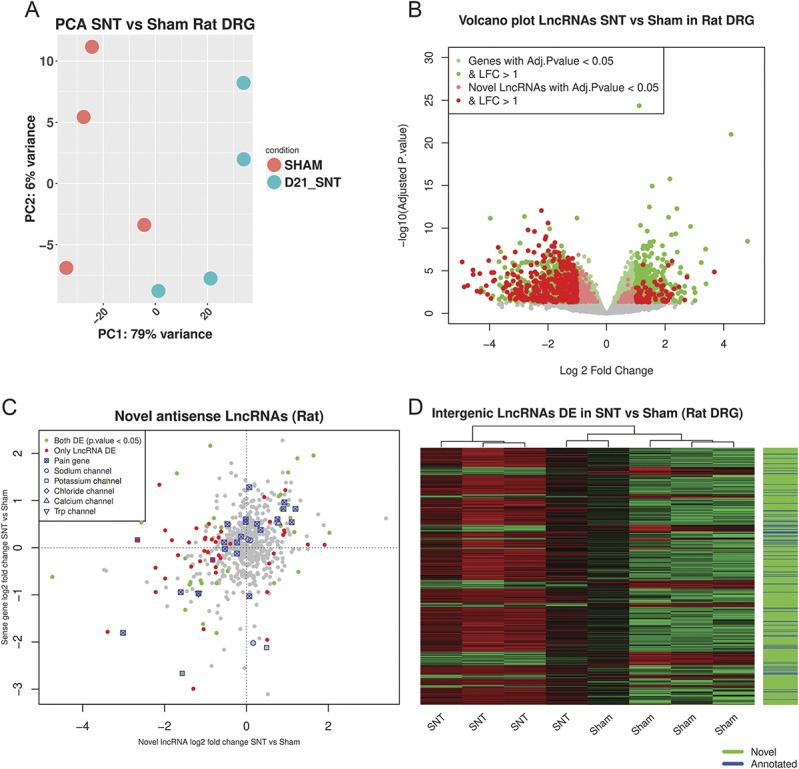
Dorsal root ganglion transcriptional response in rats after peripheral neuropathy. (A) Principal component analysis plot of samples based on the regularised log2-transformed counts of novel LncRNAs and ENSEMBL genes (first 5000 genes ranked by their SD) in the rat DRG. Axis represents PCs and shows percentage of original data's variance explained by the respective PC. (B) Volcano plot of the whole gene set (ENSEMBL annotated genes and novel LncRNAs). X-axis Log2(Fold Change), y-axis −log10(FDR adjusted *P* value). Significantly DE ENSEMBL annotated genes and novel LncRNAs are highlighted. (C) Expression plot of novel antisense LncRNAs vs the sense protein-coding gene. (D) Heatmap of novel and annotated intergenic LncRNAs DE between SNT and Sham-operated animals. DE, differentially expressed; DRG, dorsal root ganglion; FDR, false discovery rate; LncRNAs, long noncoding RNAs; PCA, principal component analysis; SNT, spinal nerve transection.

We confirmed that the expression patterns of the top 5000 ENSEMBL annotated genes and novel LncRNAs ranked by their SD could separate samples according to condition (Fig. 6A). We also observed a significant transcriptional response after nerve injury in the rat DRG, which amounts to 25.5% (4215 ENSEMBL annotated genes + novel LncRNAs) of all expressed genes (Fig. 6B, S. Data 12, available at http://doi.org/10.6084/m9.figshare.6508205). There was a significant enrichment of BP GO terms related to ion-channel transport, signal transduction, and response to mechanical stimulus in the ENSEMBL annotated DE genes (supplementary figure 5, available at http://links.lww.com/PAIN/A676). Novel LncRNAs were a substantial component of the DE genes after peripheral nerve injury (highlighted in Fig. 6B).

Five hundred forty-one of the 3169 annotated LncRNAs in rats (ENSEMBL Rnor_6.0.92) were expressed in the DRG. Out of these, 82 (16%) were significantly DE. From the 4316 putative novel LncRNAs, 708 (16.4%) were significantly DE in rats (PainNetworks, GEO GSE107180). Of the 629 novel antisense LncRNAs, 253 (40%) had an anticorrelated expression pattern to the sense gene after SNT (S. Data 13, available at http://doi.org/10.6084/m9.figshare.6508205). In total, 31 reach significance with a significant DE gene on the opposite strand. Nine of them were significantly DE on the opposite strand of a significantly DE pain gene with opposite log fold changes (supplementary table 13, available at http://links.lww.com/PAIN/A676). There were novel LncRNAs antisense of pain genes, voltage-gated potassium and sodium channels (Fig. 6C).

Six hundred fifty-four intergenic LncRNAs were found to be significantly DE in SNT vs sham, 575 of them were novel (Fig. 6D, S. Data 14, available at http://doi.org/10.6084/m9.figshare.6508205). Five of them (4 novel and 1 annotated) were adjacent to and highly correlated with pain genes (supplementary table 14, available at http://links.lww.com/PAIN/A676). Most intergenic LncRNAs were positively correlated with their closest genomic neighbour. Also, the closer they were to their closest neighbour, the stronger the correlation (supplementary figure 6, available at http://links.lww.com/PAIN/A676), both for annotated and novel LncRNAs.

#### 3.4.2. Transcriptional changes of LncRNAs in the mouse dorsal root ganglion

**Figure 7.**
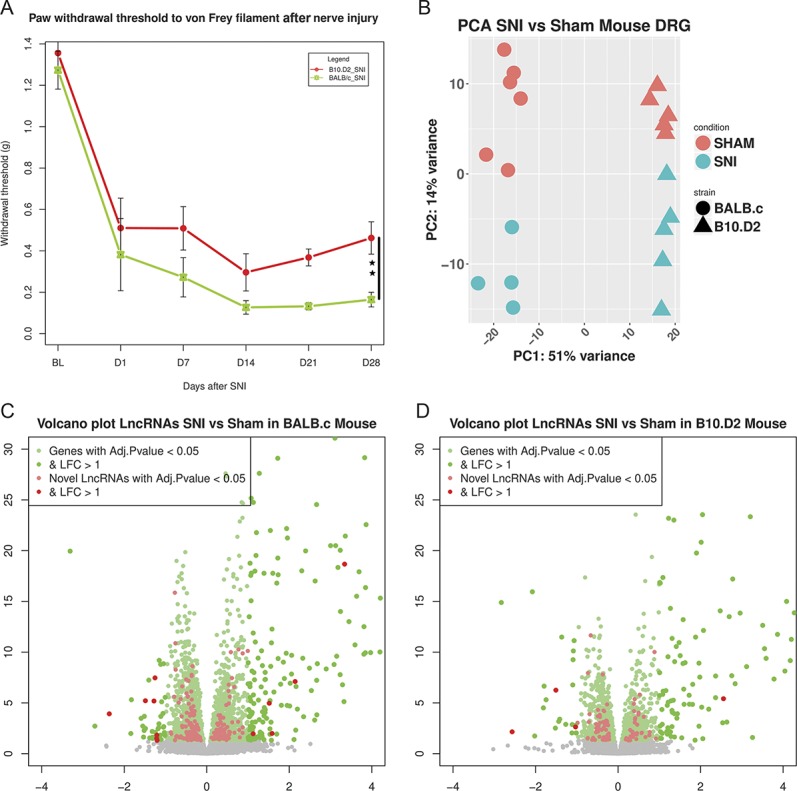
Dorsal root ganglion transcriptional response in mice after peripheral neuropathy. (A) Hind paw withdrawal thresholds to von Frey filament stimulation + SEM in grams. We calculated the area over the curve (AOC) for each strain and obtained the percentage of maximum induced hypersensitivity. Two-way ANOVA showed a significant effect of surgery and a significant interaction of strain:surgery (*P* = 0.001). One-way ANOVA between SNI groups on D28 showed significant difference in % of maximum hypersensitivity (*P* = 0.002). Black bar shows comparison between SNI groups, *P* < 0.01**. N = 12 per strain, N = 6 per SNI group. (B) Principal component analysis plot of samples based on the expression of novel LncRNAs and ENSEMBL genes (first 10,000 genes ranked by their SD) in the mouse DRG. Axis represents PCs and show percentage of original data's variance explained by the respective PC. (C and D) Volcano plots of the whole gene set for BALB/c strain (C) and B10.D2 strain (D). X-axis Log2(Fold Change), y-axis −log10(FDR adjusted *P* value). Significantly DE ENSEMBL annotated genes and novel LncRNAs are highlighted. ANOVA, analysis of variance; DE, differentially expressed; DRG, dorsal root ganglion; FDR, false discovery rate; LncRNAs, long noncoding RNAs; SNI, spared nerve injury.

Withdrawal thresholds to von Frey filament stimulation confirmed previous findings that the B10.D2 demonstrates less mechanical pain–related hypersensitivity after SNI than BALB/c^67^ (Fig. 7A). There were no significant behavioural differences between male and female mice for either strain. Principal components analysis of the expression of the top 10,000 ENSEMBL annotated genes and novel LncRNAs (ranked by SD) showed that they were able to optimally separate mouse samples according to sex, strain, and within them, condition (Fig. 7B).

Volcano plots show the range of transcriptional changes of ENSEMBL annotated genes and novel LncRNAs in mice after peripheral nerve injury (Figs. 7C and D, S. Data 15, available at http://doi.org/10.6084/m9.figshare.6508205). Novel LncRNAs were a substantial component of the DE genes following the SNI pain model (highlighted in Figs. 7C and D).

Regarding the BALB/c mouse strain, which showed significantly more pain-related hypersensitivity from day 7 onwards than the B10.D2 strain, we found significantly more DE ENSEMBL annotated genes and novel LncRNAs, 2750 compared with 1441. In comparison to rats, we found less DE genes in the mouse after nerve injury. Spinal nerve transection is a more severe injury model than SNI (in which less sensory neurons are axotomised and the injury is more distal), and so, this may reflect model severity rather than species differences.

**Figure 8.**
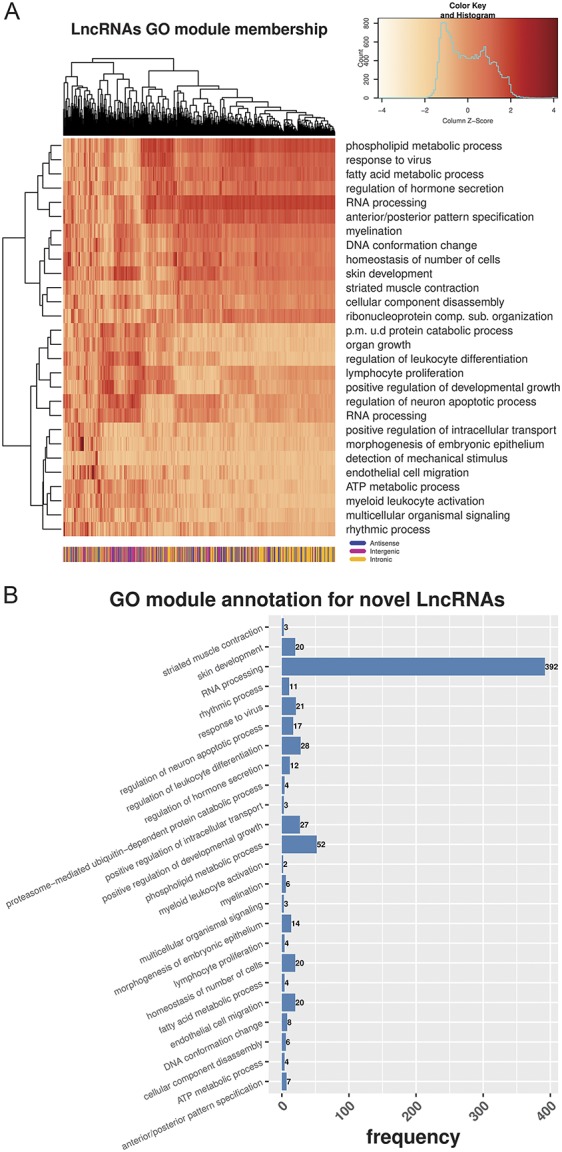
Network analysis of annotated genes and novel LncRNAs. (A) Heatmap of module membership for novel LncRNAs. Module membership quantified with absolute bicorrelation. Colours represent *z*-values of absolute bicorrelation. (B) Distribution of novel LncRNAs in modules enriched with GO terms of Biological Process (BP). GO, Gene Ontology; LncRNAs, long noncoding RNAs.

In those ENSEMBL annotated genes that were DE, we found significant GO enrichment in terms related to the nervous system, regulation of excitability, signal transduction, and response to injury (supplementary figure 7, available at http://links.lww.com/PAIN/A676). As novel LncRNAs were not assigned with GO terms, we used their expression profiling context, in an unbiased way, to gather more insights regarding their possible functional importance. Under the assumption that they may regulate gene expression either *in-cis* or *in-trans*, we further studied them in the context of modules of closely coexpressed genes and associated them with enriched GO BP terms. We first created a weighted gene coexpression network (WGCNA)^36^ and then identified modules of highly coexpressed ENSEMBL annotated genes and novel LncRNAs (scale independence and dendrogram of merged/unmerged modules in supplementary figure 8, available at http://links.lww.com/PAIN/A676). The absolute weighted bicorrelation across all samples (n = 20) was used to construct the network. An n > 15 is needed to robustly calculate expression correlations. We also calculated the representative gene (ie, eigengene) for each module, defined as the first principal component of the expression of all member genes. We then quantified the novel LncRNAs' module membership by calculating the robust weighted bicorrelation of the LncRNAs with these eigengenes. Next, we performed a GO enrichment analysis and annotated each module with its top enriched BP term, based on an overrepresentation analysis of the annotated genes. The strength of module membership for novel LncRNAs (Fig. 8A) shows highly correlated modules of LncRNAs associated with RNA-processing and some related to the nervous system, signalling, and regeneration. The vast majority of novel LncRNAs were in the module associated with RNA-processing (Fig. 8B).

Of the 7990 annotated ENSEMBL (GRCm38.87) LncRNAs, 2406 were expressed in the mouse DRG. A total of 296 LncRNAs were significantly DE in BALB/c strain SNI vs sham, 193 of them were novel. In B10.D2 strain, 146 LncRNAs, 97 of which were novel, were significantly DE (PainNetworks and GEO GSE107180). Although the absolute numbers are much smaller in comparison with annotated genes, percentages of significantly dysregulated transcripts are similar. Most of them were intergenic (S. Data 16, available at http://doi.org/10.6084/m9.figshare.6508205) and antisense (S. Data 17, available at http://doi.org/10.6084/m9.figshare.6508205).

**Figure 9.**
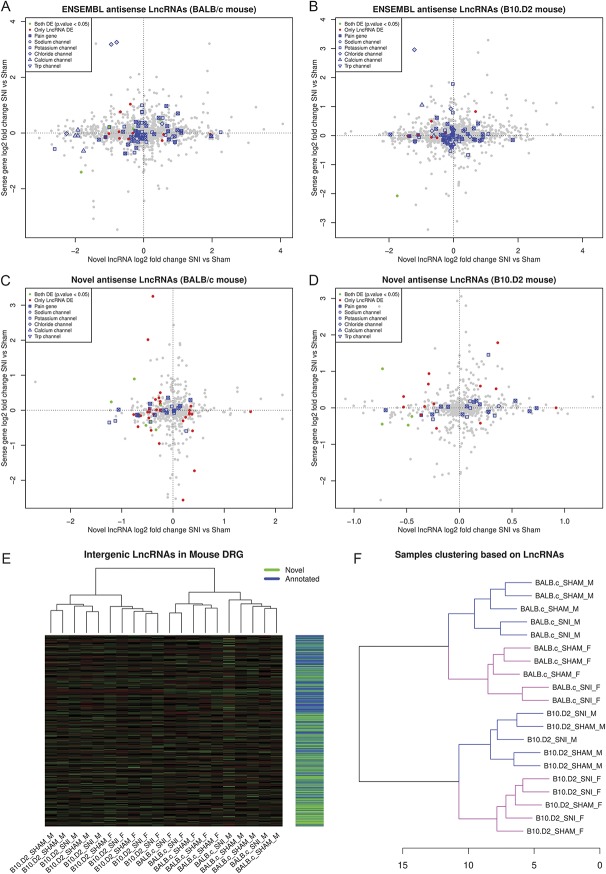
Expression patterns of LncRNAs in the mouse DRG. (A and B) Expression plot of annotated ENSEMBL antisense LncRNAs in the BALB/c mouse (A) and B10.D2 mouse (B). (C and D) Novel antisense LncRNAs vs the sense protein-coding gene in BALB/c (C) and B10.D2 (D) strains. Novel LncRNAs antisense to DE genes *Inpp1*, *Epyc*, *Kcnmb1*, *Nefl*, *Nalcn*, *Nbea*, *Ttc39aos1*, *Cgref1*, and *Zyg11b* are significantly DE. (E) Heatmap of novel and annotated intergenic LncRNAs DE between SNT and Sham-operated animals. (F) Hierarchical samples' clustering based on the expression of ENSEMBL annotated and novel LncRNAs in mice. Counts were transformed using the regularized log2 transformation; Euclidean distance was used as a dissimilarity measure and complete linkage was used for clustering. Male samples are in blue and female in pink colour. DE, differentially expressed; DRG, dorsal root ganglion; LncRNAs, long noncoding RNAs; SNI, spared nerve injury; SNT, spinal nerve transection.

Forty percent of the 1776 annotated antisense LncRNAs in mice had an anticorrelated expression pattern with their sense gene (Figs. 9A and B). 44.8% of the novel antisense LncRNAs had opposite fold changes in comparison with their protein-coding gene on the opposite strand (Figs. 9C and D). Some demonstrated the opposite expression pattern to their sense gene after nerve injury (Figs. 9A–D, supplementary table 15, available at http://links.lww.com/PAIN/A676). The significantly DE LncRNAs antisense to sodium and potassium channels are in supplementary table 16 (available at http://links.lww.com/PAIN/A676). The majority of LncRNAs antisense to potassium channels were downregulated after nerve injury and between IPSC-derived neurons and IPSC. On the other hand, although the LncRNA antisense to Scn9a was downregulated after nerve injury, all other LncRNAs antisense to sodium channels were upregulated in IPSC-derived neurons vs IPSC. In some cases, these showed an opposite expression pattern to their sense gene.

In total, 2365 intergenic LncRNAs were expressed in the mouse DRG, 1282 of them were novel, and 126 were significantly DE in mouse SNI vs Sham (Fig. 9E). Similarly to rats and humans, intergenic LncRNAs showed positive correlation with their adjacent gene (supplementary figure 9, available at http://links.lww.com/PAIN/A676). Twenty-three novel and 18 known intergenic LncRNAs were adjacent to pain genes in mice. Two of these novel intergenic LncRNAs were significantly DE and highly correlated with their adjacent pain gene (supplementary table 17, available at http://links.lww.com/PAIN/A676). In some cases, multiple novel LncRNAs related to coding genes in the same pain-related signalling pathway could be identified and were DE in mice and rats. For instance, 2 intergenic LncRNAs upstream of the opioid receptor genes, *Oprl1* and *Oprd1*, were significantly DE and highly correlated with their adjacent significantly DE gene in both species. *Oprl1* and *Oprd1* form heterodimers and appear in the same network of highly correlated genes.^54^

We also performed unsupervised clustering of samples based on the expression of all novel and annotated LncRNAs. We observed a pattern of highly sex-specific expression changes within strains and then, within sex, we had separation according to condition (Fig. 9F). This indicated that LncRNAs are dysregulated after peripheral nerve injury in a sex- and strain-dependent manner.

We selected and validated 7 representative novel LncRNAs based on the expression strength, the significance and size of the effect (log2 fold change), and the genomic context. These novel LncRNAs were all found to be significantly DE in the mouse DRG after nerve injury and among these are antisense and intergenic, table 2 (primers in supplementary table 18, primer binding in supplementary figure 10, available at http://links.lww.com/PAIN/A676).

**Table 2:**
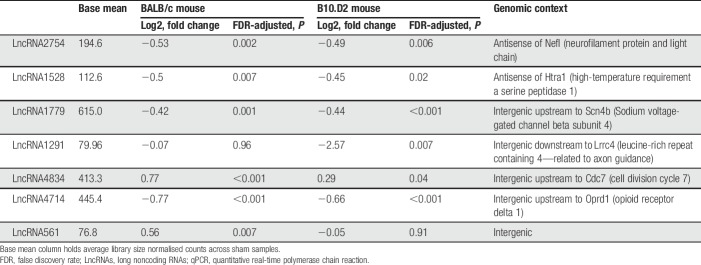
Representative qPCR validated novel LncRNAs.

**Figure 10.**
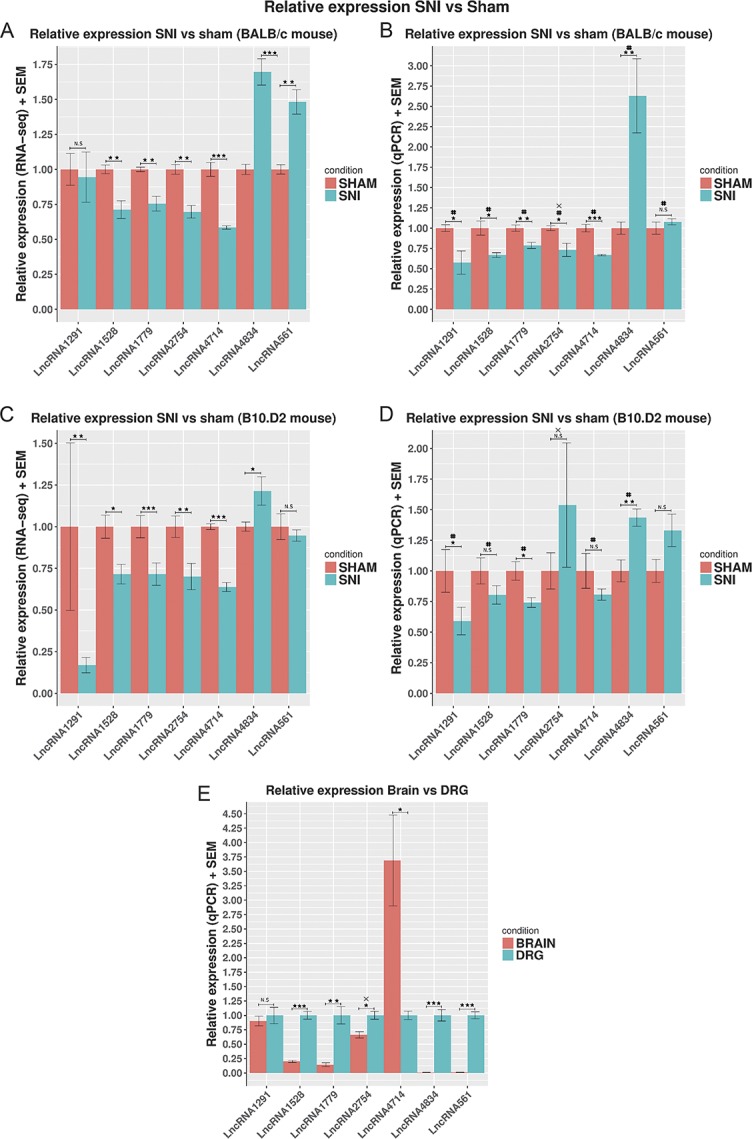
Relative expression of 7 novel LncRNAs in the SNI vs Sham mouse DRG assessed by qPCR and RNA-seq. (A and C) Relative expression assessed by RNA-seq. RNA-seq counts were normalised by the sham average and the effective library size using DESeq2. Significance as obtained by DESeq2 using the following GLM ∼ sex + strain × condition for the whole gene set of ENSEMBL annotated genes and novel LncRNAs, N = 10 per strain (6 Sham–4 SNI BALB/c, 5 Sham–5 SNI B10.D2). (B and D) Relative expression assessed by qPCR. Expression was normalized against the average expression in Sham. Significance was obtained using a 1-way ANOVA and the linear model ∼ sex + condition, N = 10 per strain. (E) Relative expression of 7 novel LncRNAs in brain vs DRG. Expression was measured by qPCR using the delta CT method. Expression was normalized against the average expression in the brain. Data are presented as mean plus SEM. *P* < 0.05*, *P* < 0.01**, and *P* < 0.001***, same direction of change between RNA-seq and qPCR #. X indicates strand-specific RT-PCR, N = 8 for B10.D2 (5 Sham and 3 SNI). ANOVA, analysis of variance; DRG, dorsal root ganglion; LncRNAs, long noncoding RNAs; qPCR, quantitative real-time polymerase chain reaction; SNI, spared nerve injury.

Quantitative real-time polymerase chain reaction confirmed the expression of these novel LncRNAs in the mouse DRG and validated their dysregulation after nerve injury, Figure 10. With the exception of 1 LncRNA, these all demonstrated higher expression in the DRG compared with brain (Fig. 10E). This comprehensive study^47^ showed that upstream of genes where these LncRNAs lie, there were no previously unannotated lengthened 3′ UTRs. With the exception of LncRNA4714, upstream of Oprd1, where the multiexonic transcript we have identified is much longer than any predicted elongated UTR. When studying the changes in LncRNA expression evoked by SNI in B10.D2 and BALB/c mouse strains, there was in general good agreement between RNA-seq and qPCR findings. We also found that in all cases, where RNA-sequencing showed significant dysregulation, qPCR confirmed the result (Fig. 10).

## 4. Discussion

We used high-depth RNA sequencing and a dedicated bioinformatic pipeline to identify thousands of known and putative novel LncRNAs expressed in the mouse and rat DRG and human IPSC–derived sensory neurons. Many of these LncRNAs were antisense or adjacent to known pain and ion-channel genes. A significant proportion demonstrated selective expression in DRG neuron subtypes. Novel LncRNAs were DE after peripheral nerve injury in a species- and strain-dependent manner, including novel antisense LncRNAs with opposite transcriptional changes to their sense genes and intergenic LncRNAs highly correlated with their adjacent gene. Thus, LncRNAs that have been a relatively unexplored part of the DRG transcriptome demonstrate remarkable complexity in terms of their relationship to genes known to impact on sensory function, DRG cell-type–specific expression, and the transcriptional response to nerve injury.

LncRNAs have been shown to be: smaller than protein-coding genes, spliced, biexonic, transcribed as independent transcription units, expressed at a lower level than protein-coding genes, and show highly spatially constrained correlation with their antisense and adjacent genes.^17,69,81^ These characteristics are highly consistent with the antisense and intergenic LncRNAs that we identified in this study as being expressed in the DRG.

No previous attempts have comprehensively determined LncRNA expression within the DRG. Previous studies have undertaken a candidate gene approach,^32,86^ whereas others have used a high-throughput approach such as RNA-seq but have only interrogated the expression of previously annotated LncRNAs.^24,30,75^ As we have shown, this strategy will miss thousands of unannotated and potentially important LncRNAs. In fact, 8% of genes within the Pain Genes Database in the mouse, 16% in the rats, and 7% in human IPSC were found to have an antisense or neighbouring intergenic LncRNA. Furthermore, LncRNAs antisense to ion channels were not limited to KCNA2 and SCN9A, but could also be identified to other sodium, potassium, calcium, and also TRP channels, all of which have key roles in modulating sensory neuron function. As with all computational predictions, this study has the limitation of the possible inclusion of false positives in our list of novel LncRNAs; however, we have validated the expression of a number of novel LncRNAs using the independent technique of qPCR.

It is known that LncRNAs can be highly tissue- and cell-type–specific.^57,68^ Dorsal root ganglion cells are heterogeneous in terms of their physiology, anatomy, and connectivity; recent single-cell analysis shows how gene expression relates to such specialised features but also reveals even greater complexity in DRG subtypes based on molecular profiling.^38,70^ We showed that both novel and annotated LncRNAs demonstrate selective expression within sensory neuron subtypes. As such, identification of LncRNAs may help in the molecular classification of DRG cells. Moreover, both novel and annotated LncRNAs were significantly enriched in neuron-subtype–specific genes vs protein-coding genes. The functional specialisation of different neuron subtypes will principally arise due to the neuron subtype–dependent expression of protein-coding genes; however, such expression may be sculpted by LncRNAs.

We have also defined LncRNAs expressed in human IPSC-derived sensory neurons. Advantages of studying LncRNA expression in this model (rather than, eg, cadaveric human DRG) is the ready source of high-quality RNA from a pure sensory neuronal population (with very few contaminating Schwann cells) and the ability to compare expression to the parent IPSC line. Disadvantages include the fact that although subpopulations of sensory neurons exist in these cultures, these are not as diverse as native DRG cells and are less mature given the lack of target interactions.

We identified almost 2000 previously annotated and over 2000 novel putative LncRNAs, which were DE when comparing IPSC-derived sensory neurons and parent IPSC lines. These demonstrated many characteristics of the LncRNAs expressed in the rodent DRG. Twenty-nine percent of genes in the Pain Genes Database had a potentially relevant relationship to a novel or annotated LncRNA either antisense LncRNAs on the opposite strand or adjacent to an intergenic LncRNA. Interestingly, some of these LncRNAs may have a role in shaping the expression of the sense pain gene during differentiation, as both novel and annotated, DE antisense LncRNAs demonstrated anticorrelated expression changes to their DE sense pain gene. We also identified human LncRNAs that were antisense to voltage-gated ion channel genes. It has been shown that many LncRNAs overlap genome-wide association studies' traits, and LncRNAs overlapping trait-associated SNPs are specifically expressed in cell types relevant to the traits.^28^ The identification of human LncRNAs expressed in IPSC-derived sensory neurons may therefore enable investigation of the genetic basis of chronic pain states in humans, especially under conditions of neuropathic pain wherein maladaptive plasticity in the DRG is an important pathophysiological driver. A recent study has mapped eQTLs in the human DRG,^52^ and a number of SNPs that impacted on gene expression were found to be within annotated LncRNAs. In our data, 9 LncRNAs expressed in IPSC-derived sensory neurons were overlapped by eQTLs. Three of the novel and 3 of the known were also DE in IPSC-derived neurons vs IPSC. Dorsal root ganglion eQTLs identified by Ref. 52 were found among hits in numerous genome-wide association studies. Such studies highlight the utility in identifying LncRNAs, in this case by providing explanatory power as to how an SNP linked to complex disorders may impact on gene expression and phenotype. As more tissue becomes available for RNA-seq, it will also be possible to extend LncRNA discovery to the postmortem human DRG.^60^

Nerve injury is known to elicit remarkable transcriptional changes within the DRG, which has deleterious consequences such as neuropathic pain, but in certain contexts can also be adaptive; eg, by promoting nerve repair.^51^ We investigated the expression of LncRNAs in Wistar rats after SNT and in 2 different mouse strains after SNI. The BALB/c strain was previously shown to develop greater levels of mechanical hypersensitivity after SNI vs the B10.D2 strain.^67^ Hundreds of LncRNAs were DE in both rats and mice after nerve injury. We found that more protein-coding genes, annotated LncRNAs, and novel LncRNAs were DE in the high pain BALB/c strain compared with the low pain B10.D2 strain. Most of the variance of the LncRNAs' expression in our data set was between strains and not conditions; namely, LncRNAs demonstrated strain-dependent expression plasticity after nerve injury. Twenty-seven percent of LncRNAs were significantly DE between mouse strains compared with 12.2% for ENSEMBL protein-coding genes. Considering the rapidly increasing literature on sex differences in pain processing^48,66^ that there were sex-dependent effects on LncRNA expression.

Weighted gene coexpression network analysis revealed that novel LncRNAs in the mouse DRG were in network modules related to RNA-processing and some of them in modules related to myelination, development, and regeneration of the nervous system, immune response, and signalling. This could indicate that these LncRNAs function together with genes acting as transcriptional regulators associated with posttranscriptional modifications. This enrichment is different from that observed in the ENSEMBL annotated gene set, where terms related to axon guidance, ion channel transport, regulation of synapse assembly and of neuron apoptotic process, nervous system development, and memory were significantly enriched. These findings are similar to known biological processes enriched after nerve injury^83,84^ and in pain genes.^41^

Enrichment in biological processes of splicing, mRNA processing, and polyadenylation (ie, parent, child, and related terms to RNA-processing) has however been reported.^82,83^ Our finding that the expression of novel LncRNAs changes together with annotated genes regulating RNA-processing is consistent with known mechanisms by which LncRNAs alter gene expression (discussed below).

LncRNAs can regulate expression in *cis* and in *trans*,^33^ but the genomic context of LncRNAs is important for both antisense transcripts regulating the gene on the sense strand or intergenic LncRNAs. Intergenic and antisense LncRNAs, which tend to lie in genomic regions populated with genes,^31^ have correlated expression patterns with their adjacent genes and may regulate gene expression (Fig. 4).^58^ We found that the shorter the distance, the stronger the correlation in all species, both for known and annotated LncRNAs. However, the network analysis we performed allowed us to identify in an unbiased way modules of highly correlated genes and LncRNAs across all samples. Thus, these LncRNAs are putative both *in-cis* and *in-trans* regulators. Regarding the relation of LncRNAs with their genomic context, we found that more than 45% of antisense LncRNAs had anticorrelated—opposite expression changes in respect to the sense gene, whereas only 10% of intergenic LncRNAs had negative correlation with the expression of their closest genomic neighbour. Twelve pairs of antisense LncRNA/sense gene with opposite LFCs reached significance between pain models and control animals. These antisense LncRNAs fit into the paradigm of *Kcna2* antisense,^86^ which silences the gene on the opposite strand. LncRNAs are also known for regulating clusters of imprinted genes or close genes such as the *Hoxd* cluster.^43,78^ One of these LncRNAs, HAGLR, was the most upregulated and stronger expressed LncRNA in human IPSC–derived neurons and was also found by in situ hybridization to be expressed in the mouse DRG and significantly downregulated in both mouse strains after nerve injury (supplementary table 12, available at http://links.lww.com/PAIN/A676).

We used qPCR to validate the expression of a number of novel LncRNAs in close genomic relationship to protein-coding genes of relevance to sensory function: A novel LncRNA antisense of Nefl gene implicated in the Charcot–Marie–Tooth disease,^85^ and an intergenic LncRNA close to and highly correlated with the pain gene Oprd1 was found DE and validated with qPCR. We also describe a novel intergenic LncRNA in close proximity to the Lrrc4 gene that relates to axon guidance and synapse organisation,^16,74^ and finally, a significantly DE intergenic LncRNA was validated close to the voltage-gated sodium channel subunit Scn4b. The majority of LncRNAs validated by qPCR were found to be more highly expressed in the DRG than brain. LncRNAs are putative therapeutic agents that could regulate the expression of target genes related to disease.^71^ Although application of such therapeutics to pain would need to overcome the hurdle of delivering therapeutics to DRG cell bodies.

In summary, we have provided a resource, in which we have defined LncRNA expression within the DRG across species. We show that marked changes in LncRNA expression occur after nerve injury and during sensory neuron differentiation. LncRNAs' expression is DRG subtype–specific, and there is often highly spatially constrained correlation/anticorrelation with their antisense and adjacent genes.

## Conflict of interest statement

The authors have no conflict of interest to declare.

## Supplementary Material

SUPPLEMENTARY MATERIAL

## Appendix A. Supplemental digital content

Supplemental digital content associated with this article can be found online at http://links.lww.com/PAIN/A676.

## References

[1] Abraira VE Ginty DD The sensory neurons of touch. Neuron 2013;79:618–39. 23972592 10.1016/j.neuron.2013.07.051 PMC3811145

[2] Alexa A Rahnenfuhrer J topGO: topGO: enrichment analysis for gene ontology. 2010 www.bioconductor.org. In R package version 3.4.4.

[3] Anders S Pyl PT Huber W HTSeq—a Python framework to work with high-throughput sequencing data. Bioinformatics 2015;31:166–9. 25260700 10.1093/bioinformatics/btu638 PMC4287950

[4] Basbaum AI Bautista DM Scherrer G Julius D Cellular and molecular mechanisms of pain. Cell 2009;139:267–84. 19837031 10.1016/j.cell.2009.09.028 PMC2852643

[5] van Brakel JP Algorithm—peak signal detection in realtime timeseries data—stack overflow. 2014 Available at: https://stackoverflow.com/questions/22583391/peak-signal-detection-in-realtime-timeseries-data/22640362. Accessed May 1, 2018.

[6] Brown JB Boley N Eisman R May GE Stoiber MH Duff MO Booth BW Wen J Park S Suzuki AM Wan KH Yu C Zhang D Carlson JW Cherbas L Eads BD Miller D Mockaitis K Roberts J Davis CA Frise E Hammonds AS Olson S Shenker S Sturgill D Samsonova AA Weiszmann R Robinson G Hernandez J Andrews J Bickel PJ Carninci P Cherbas P Gingeras TR Hoskins RA Kaufman TC Lai EC Oliver B Perrimon N Graveley BR Celniker SE Diversity and dynamics of the drosophila transcriptome. Nature 2014;512:393–399. 24670639 10.1038/nature12962 PMC4152413

[7] Cabili MN Trapnell C Goff L Koziol M Tazon-Vega B Regev A Rinn JL Integrative annotation of human large intergenic noncoding RNAs reveals global properties and specific subclasses. Genes Dev 2011;25:1915–27. 21890647 10.1101/gad.17446611 PMC3185964

[8] Chambers SM Qi Y Mica Y Lee G Zhang XJ Niu L Bilsland J Cao L Stevens E Whiting P Shi SH Studer L Combined small-molecule inhibition accelerates developmental timing and converts human pluripotent stem cells into nociceptors. Nat Biotechnol 2012;30:715–20. 22750882 10.1038/nbt.2249 PMC3516136

[9] Chandran V Coppola G Nawabi H Omura T Versano R Huebner EA Zhang A Costigan M Yekkirala A Barrett L Blesch A Michaelevski I Davis-Turak J Gao F Langfelder P Horvath S He Z Benowitz L Fainzilber M Tuszynski M Woolf CJ Geschwind DH A systems-level analysis of the peripheral nerve intrinsic axonal growth program. Neuron 2016;89:956–70. 26898779 10.1016/j.neuron.2016.01.034 PMC4790095

[10] Chaplan SR Bach FW Pogrel JW Chung JM Yaksh TL Quantitative assessment of tactile allodynia in the rat paw. J Neurosci Methods 1994;53:55–63. 7990513 10.1016/0165-0270(94)90144-9

[11] Clark AJ Kaller MS Galino J Willison HJ Rinaldi S Bennett DLH Co-cultures with stem cell-derived human sensory neurons reveal regulators of peripheral myelination. Brain J Neurol 2017;140:898–913. 10.1093/brain/awx012 PMC5637940 28334857

[12] Colloca L Ludman T Bouhassira D Baron R Dickenson AH Yarnitsky D Freeman R Truini A Attal N Finnerup NB Eccleston C Kalso E Bennett DL Dworkin RH Raja SN Neuropathic pain. Nat Rev Dis Primer 2017;3:17002. 10.1038/nrdp.2017.2 PMC5371025 28205574

[13] Costigan M Befort K Karchewski L Griffin RS D'Urso D Allchorne A Sitarski J Mannion JW Pratt RE Woolf CJ Replicate high-density rat genome oligonucleotide microarrays reveal hundreds of regulated genes in the dorsal root ganglion after peripheral nerve injury. BMC Neurosci 2002;3:16. 12401135 10.1186/1471-2202-3-16 PMC139981

[14] Dawes JM Weir GA Middleton SJ Patel R Chisholm KI Pettingill P Peck LJ Sheridan J Shakir A Jacobson L Gutierrez-Mecinas M Galino J Walcher J Kühnemund J Kuehn H Sanna MD Lang B Clark AJ Themistocleous AC Iwagaki N West SJ Werynska K Carroll L Trendafilova T Menassa DA Giannoccaro MP Coutinho E Cervellini I Tewari D Buckley C Leite MI Wildner H Zeilhofer HU Peles E Todd AJ McMahon SB Dickenson AH Lewin GR Vincent A Bennett DL Immune or genetic-mediated disruption of CASPR2 causes pain hypersensitivity due to enhanced primary afferent excitability. Neuron 2018;97:806–22.e10. 29429934 10.1016/j.neuron.2018.01.033 PMC6011627

[15] Decosterd I Woolf CJ Spared nerve injury: an animal model of persistent peripheral neuropathic pain. PAIN 2000;87:149–58. 10924808 10.1016/S0304-3959(00)00276-1

[16] DeNardo LA de Wit J Otto-Hitt S Ghosh A NGL-2 regulates input-specific synapse development in CA1 pyramidal neurons. Neuron 2012;76:762–75. 23177961 10.1016/j.neuron.2012.10.013 PMC7566585

[17] Derrien T Johnson R Bussotti G Tanzer A Djebali S Tilgner H Guernec G Martin D Merkel A Knowles DG Lagarde J Veeravalli L Ruan X Ruan Y Lassmann T Carninci P Brown JB Lipovich L Gonzalez JM Thomas M Davis CA Shiekhattar R Gingeras TR Hubbard TJ Notredame C Harrow J Guigó R The GENCODE v7 catalog of human long noncoding RNAs: analysis of their gene structure, evolution, and expression. Genome Res 2012;22:1775–89. 22955988 10.1101/gr.132159.111 PMC3431493

[18] Dobin A Davis CA Schlesinger F Drenkow J Zaleski C Jha S Batut P Chaisson M Gingeras TR STAR: ultrafast universal RNA-seq aligner. Bioinformatics 2013;29:15–21. 23104886 10.1093/bioinformatics/bts635 PMC3530905

[19] ENCODE consortium. ENCODE—guidelines and best practices for RNA-Seq. 2016 Available at: https://www.encodeproject.org/documents/cede0cbe-d324-4ce7-ace4-f0c3eddf5972/@@download/attachment/ENCODE%20Best%20Practices%20for%20RNA_v2.pdf. Accessed October 12, 2017.

[20] FANTOM5 CAGE profiles of human and mouse samples scientific data. Available at: https://www.nature.com/articles/sdata2017112. Accessed October 12, 2017. 10.1038/sdata.2017.112 PMC5574368 28850106

[21] Gentleman RC Carey VJ Bates DM Bolstad B Dettling M Dudoit S Ellis B Gautier L Ge Y Gentry J Hornik K Hothorn T Huber W Iacus S Irizarry R Leisch F Li C Maechler M Rossini AJ Sawitzki G Smith C Smyth G Tierney L Yang JY Zhang J Bioconductor: open software development for computational biology and bioinformatics. Genome Biol 2004;5:R80. 15461798 10.1186/gb-2004-5-10-r80 PMC545600

[22] Gerstein MB Rozowsky J Yan KK Wang D Cheng C Brown JB Davis CA Hillier L Sisu C Li JJ Pei B Harmanci AO Duff MO Djebali S Alexander RP Alver BH Auerbach R Bell K Bickel PJ Boeck ME Boley NP Booth BW Cherbas L Cherbas P Di C Dobin A Drenkow J Ewing B Fang G Fastuca M Feingold EA Frankish A Gao G Good PJ Guigó R Hammonds A Harrow J Hoskins RA Howald C Hu L Huang H Hubbard TJP Huynh C Jha S Kasper D Kato M Kaufman TC Kitchen RR Ladewig E Lagarde J Lai E Leng J Lu Z MacCoss M May G McWhirter R Merrihew G Miller DM Mortazavi A Murad R Oliver B Olson S Park PJ Pazin MJ Perrimon N Pervouchine D Reinke V Reymond A Robinson G Samsonova A Saunders GI Schlesinger F Sethi A Slack FJ Spencer WC Stoiber MH Strasbourger P Tanzer A Thompson OA Wan KH Wang G Wang H Watkins KL Wen J Wen K Xue C Yang L Yip K Zaleski C Zhang Y Zheng H Brenner SE Graveley BR Celniker SE Gingeras TR Waterston R Comparative analysis of the transcriptome across distant species. Nature 2014;512:445–8. 25164755 10.1038/nature13424 PMC4155737

[23] Ghazalpour A Doss S Zhang B Wang S Plaisier C Castellanos R Brozell A Schadt EE Drake TA Lusis AJ Horvath S Integrating genetic and network analysis to characterize genes related to mouse weight. PLOS Genet 2006;2:e130. 16934000 10.1371/journal.pgen.0020130 PMC1550283

[24] Gong L Wu J Zhou S Wang Y Qin J Yu B Gu X Yao C Global analysis of transcriptome in dorsal root ganglia following peripheral nerve injury in rats. Biochem Biophys Res Commun 2016;478:206–12. 27450809 10.1016/j.bbrc.2016.07.067

[25] Guo T Mandai K Condie BG Wickramasinghe SR Capecchi MR Ginty DD An evolving NGF-Hoxd1 signaling pathway mediates development of divergent neural circuits in vertebrates. Nat Neurosci 2011;14:31–6. 21151121 10.1038/nn.2710 PMC3180918

[26] Han TW Jan LY Making antisense of pain. Nat Neurosci 2013;16:986–7. 23887131 10.1038/nn.3475

[27] Harrow J Frankish A Gonzalez JM Tapanari E Diekhans M Kokocinski F Aken BL Barrell D Zadissa A Searle S Barnes I Bignell A Boychenko V Hunt T Kay M Mukherjee G Rajan J Despacio-Reyes G Saunders G Steward C Harte R Lin M Howald C Tanzer A Derrien T Chrast J Walters N Balasubramanian S Pei B Tress M Rodriguez JM Ezkurdia I van Baren J Brent M Haussler D Kellis M Valencia A Reymond A Gerstein M Guigó R Hubbard TJ GENCODE: the reference human genome annotation for the ENCODE Project. Genome Res 2012;22:1760–74. 22955987 10.1101/gr.135350.111 PMC3431492

[28] Hon CC Ramilowski JA Harshbarger J Bertin N Rackham OJL Gough J Denisenko E Schmeier S Poulsen TM Severin J Lizio M Kawaji H Kasukawa T Itoh M Burroughs AM Noma S Djebali S Alam T Medvedeva YA Testa AC Lipovich L Yip CW Abugessaisa I Mendez M Hasegawa A Tang D Lassmann T Heutink P Babina M Wells CA Kojima S Nakamura Y Suzuki H Daub CO de Hoon MJL Arner E Hayashizaki Y Carninci P Forrest ARR An atlas of human long non-coding RNAs with accurate 5’ ends. Nature 2017;543:199–204. 28241135 10.1038/nature21374 PMC6857182

[29] Ilott NE Ponting CP Predicting long non-coding RNAs using RNA sequencing. Methods 2013;63:50–9. 23541739 10.1016/j.ymeth.2013.03.019

[30] Jiang BC Sun WX He LN Cao DL Zhang ZJ Gao YJ Identification of lncRNA expression profile in the spinal cord of mice following spinal nerve ligation-induced neuropathic pain. Mol Pain 2015;11:43. 26184882 10.1186/s12990-015-0047-9 PMC4504460

[31] Kapusta A Feschotte C Volatile evolution of long noncoding RNA repertoires: mechanisms and biological implications. Trends Genet 2014;30:439–52. 25218058 10.1016/j.tig.2014.08.004 PMC4464757

[32] Koenig J Werdehausen R Linley JE Habib AM Vernon J Lolignier S Eijkelkamp N Zhao J Okorokov AL Woods CG Wood JN Cox JJ Regulation of Nav1.7: a conserved SCN9A natural antisense transcript expressed in dorsal root ganglia. PLoS One 2015;10:e0128830. 26035178 10.1371/journal.pone.0128830 PMC4452699

[33] Kornienko AE Guenzl PM Barlow DP Pauler FM Gene regulation by the act of long non-coding RNA transcription. BMC Biol 2013;11:59. 23721193 10.1186/1741-7007-11-59 PMC3668284

[34] LaCroix-Fralish ML Austin JS Zheng FY Levitin DJ Mogil JS Patterns of pain: meta-analysis of microarray studies of pain. PAIN 2011;152:1888–98. 21561713 10.1016/j.pain.2011.04.014

[35] Lacroix-Fralish ML Ledoux JB Mogil JS The Pain Genes Database: an interactive web browser of pain-related transgenic knockout studies. PAIN 2007;131:3.e1–4. 17574758 10.1016/j.pain.2007.04.041

[36] Langfelder P Horvath S WGCNA: an R package for weighted correlation network analysis. BMC Bioinformatics 2008;9:559. 19114008 10.1186/1471-2105-9-559 PMC2631488

[37] Lee J Hong W Cho M Sim M Lee D Ko Y Kim J Synteny Portal: a web-based application portal for synteny block analysis. Nucleic Acids Res 2016;44:W35–40. 27154270 10.1093/nar/gkw310 PMC4987893

[38] Li CL Li KC Wu D Chen Y Luo H Zhao JR Wang SS Sun MM Lu YJ Zhong YQ Hu XY Hou R Zhou BB Bao L Xiao HS Zhang X Somatosensory neuron types identified by high-coverage single-cell RNA-sequencing and functional heterogeneity. Cell Res 2016;26:83–102. 26691752 10.1038/cr.2015.149 PMC4816136

[39] Li H Handsaker B Wysoker A Fennell T Ruan J Homer N Marth G Abecasis G Durbin R; 1000 Genome Project Data Processing Subgroup. The sequence alignment/map format and SAMtools. Bioinformatics 2009;25:2078–9. 19505943 10.1093/bioinformatics/btp352 PMC2723002

[40] Lizio M Harshbarger J Shimoji H Severin J Kasukawa T Sahin S Abugessaisa I Fukuda S Hori F Ishikawa-Kato S Mungall CJ Arner E Baillie JK Bertin N Bono H de Hoon M Diehl AD Dimont E Freeman TC Fujieda K Hide W Kaliyaperumal R Katayama T Lassmann T Meehan TF Nishikata K Ono H Rehli M Sandelin A Schultes EA ‘t Hoen PA Tatum Z Thompson M Toyoda T Wright DW Daub CO Itoh M Carninci P Hayashizaki Y Forrest AR Kawaji H Gateways to the FANTOM5 promoter level mammalian expression atlas. Genome Biol 2015;16:22. 25723102 10.1186/s13059-014-0560-6 PMC4310165

[41] Lötsch J Doehring A Mogil JS Arndt T Geisslinger G Ultsch A Functional genomics of pain in analgesic drug development and therapy. Pharmacol Ther 2013;139:60–70. 23567662 10.1016/j.pharmthera.2013.04.004

[42] Love MI Huber W Anders S Moderated estimation of fold change and dispersion for RNA-seq data with DESeq2. Genome Biol 2014;15:550. 25516281 10.1186/s13059-014-0550-8 PMC4302049

[43] Maamar H Cabili MN Rinn J Raj A linc-HOXA1 is a noncoding RNA that represses Hoxa1 transcription in cis. Genes Dev 2013;27:1260–71. 23723417 10.1101/gad.217018.113 PMC3690399

[44] Marques AC Ponting CP Intergenic lncRNAs and the evolution of gene expression. Curr Opin Genet Dev 2014;27:48–53. 24852186 10.1016/j.gde.2014.03.009

[45] Martin JA Wang Z Next-generation transcriptome assembly. Nat Rev Genet 2011;12:671–82. 21897427 10.1038/nrg3068

[46] Meyer LR Zweig AS Hinrichs AS Karolchik D Kuhn RM Wong M Sloan CA Rosenbloom KR Roe G Rhead B Raney BJ Pohl A Malladi VS Li CH Lee BT Learned K Kirkup V Hsu F Heitner S Harte RA Haeussler M Guruvadoo L Goldman M Giardine BM Fujita PA Dreszer TR Diekhans M Cline MS Clawson H Barber GP Haussler D Kent WJ The UCSC genome browser database: extensions and updates 2013. Nucleic Acids Res 2012;41:D64–9. 23155063 10.1093/nar/gks1048 PMC3531082

[47] Miura P Shenker S Andreu-Agullo C Westholm JO Lai EC Widespread and extensive lengthening of 3′ UTRs in the mammalian brain. Genome Res 2013;23:812–25. 23520388 10.1101/gr.146886.112 PMC3638137

[48] Mogil JS Sex differences in pain and pain inhibition: multiple explanations of a controversial phenomenon. Nat Rev Neurosci 2012;13:859–66. 23165262 10.1038/nrn3360

[49] Morgan M Falcon S Gentleman R GSEABase: gene set enrichment data structures and methods, 2017 www.bioconductor.org. In R package 3.4.4.

[50] Morgan M Obenchain V Lang M Thompson R BiocParallel: bioconductor facilities for parallel evaluation. 2017 Available at: https://github.com/Bioconductor/BiocParallel. Accessed October 12, 2017.

[51] O’Donovan KJ Intrinsic axonal growth and the drive for regeneration. Front Neurosci 2016;10:486. 27833527 10.3389/fnins.2016.00486 PMC5081384

[52] Parisien M Khoury S Chabot-Doré AJ Sotocinal SG Slade GD Smith SB Fillingim RB Ohrbach R Greenspan JD Maixner W Mogil JS Belfer I Diatchenko L Effect of human genetic variability on gene expression in dorsal root ganglia and association with pain phenotypes. Cell Rep 2017;19:1940–52. 28564610 10.1016/j.celrep.2017.05.018 PMC5524461

[53] Perkins JR Antunes-Martins A Calvo M Grist J Rust W Schmid R Hildebrandt T Kohl M Orengo C McMahon SB Bennett DL A comparison of RNA-seq and exon arrays for whole genome transcription profiling of the L5 spinal nerve transection model of neuropathic pain in the rat. Mol Pain 2014;10:7. 24472155 10.1186/1744-8069-10-7 PMC4021616

[54] Perkins JR Lees J Antunes-Martins A Diboun I McMahon SB Bennett DLH Orengo C PainNetworks: a web-based resource for the visualisation of pain-related genes in the context of their network associations. PAIN 2013;154:2586–e1–12. 24036287 10.1016/j.pain.2013.09.003 PMC3863956

[55] Pesole G Liuni S Grillo G Licciulli F Mignone F Gissi C Saccone C UTRdb and UTRsite: specialized databases of sequences and functional elements of 5′ and 3′ untranslated regions of eukaryotic mRNAs. Nucleic Acids Res 2002;30:335–40. 11752330 10.1093/nar/30.1.335 PMC99102

[56] Petryszak R Keays M Tang YA Fonseca NA Barrera E Burdett T Füllgrabe A Fuentes AMP Jupp S Koskinen S Mannion O Huerta L Megy K Snow C Williams E Barzine M Hastings E Weisser H Wright J Jaiswal P Huber W Choudhary J Parkinson HE Brazma A Expression Atlas update—an integrated database of gene and protein expression in humans, animals and plants. Nucleic Acids Res 2016;44:D746–52. 26481351 10.1093/nar/gkv1045 PMC4702781

[57] Ponjavic J Ponting CP Lunter G Functionality or transcriptional noise? Evidence for selection within long noncoding RNAs. Genome Res 2007;17:556–65. 17387145 10.1101/gr.6036807 PMC1855172

[58] Ponting CP Oliver PL Reik W Evolution and functions of long noncoding RNAs. Cell 2009;136:629–41. 19239885 10.1016/j.cell.2009.02.006

[59] R Core Team. R: a language and environment for statistical computing. Vienna: R Foundation for Statistical Computing, 2017 Available at: https://www.R-project.org/. Accessed October 12, 2017.

[60] Ray P Torck A Quigley L Wangzhou A Neiman M Rao C Lam T Kim JY Kim TH Zhang MQ Dussor G Price TJ Comparative transcriptome profiling of the human and mouse dorsal root ganglia: an RNA-seq-based resource for pain and sensory neuroscience research. PAIN 2018;159:1325–1345. 29561359 10.1097/j.pain.0000000000001217 PMC6008200

[61] Rigaud M Gemes G Barabas ME Chernoff DI Abram SE Stucky CL Hogan QH Species and strain differences in rodent sciatic nerve anatomy: implications for studies of neuropathic pain. PAIN 2008;136:188–201. 18316160 10.1016/j.pain.2008.01.016 PMC2700063

[62] Savell KE Gallus NVN Simon RC Brown JA Revanna JS Osborn MK Song EY O’Malley JJ Stackhouse CT Norvil A Gowher H Sweatt JD Day JJ Extra-coding RNAs regulate neuronal DNA methylation dynamics. Nat Commun 2016;7:12091. 27384705 10.1038/ncomms12091 PMC4941050

[63] Willis WD Jr Sensory mechanisms of the spinal cord—volume 1. Springer Available at: http://www.springer.com/cn/book/9780306480331. Accessed July 13, 2017.

[64] Shields SD Eckert WA Basbaum AI Spared nerve injury model of neuropathic pain in the mouse: a behavioral and anatomic analysis. J Pain 2003;4:465–70. 14622667 10.1067/s1526-5900(03)00781-8

[65] Shiraki T Kondo S Katayama S Waki K Kasukawa T Kawaji H Kodzius R Watahiki A Nakamura M Arakawa T Fukuda S Sasaki D Podhajska A Harbers M Kawai J Carninci P Hayashizaki Y Cap analysis gene expression for high-throughput analysis of transcriptional starting point and identification of promoter usage. Proc Natl Acad Sci U S A 2003;100:15776–81. 14663149 10.1073/pnas.2136655100 PMC307644

[66] Sorge RE Mapplebeck JCS Rosen S Beggs S Taves S Alexander JK Martin LJ Austin JS Sotocinal SG Chen D Yang M Shi XQ Huang H Pillon NJ Bilan PJ Tu Y Klip A Ji RR Zhang J Salter MW Mogil JS Different immune cells mediate mechanical pain hypersensitivity in male and female mice. Nat Neurosci 2015;18:1081–3. 26120961 10.1038/nn.4053 PMC4772157

[67] Sorge RE Trang T Dorfman R Smith SB Beggs S Ritchie J Austin JS Zaykin DV Meulen HV Costigan M Herbert TA Yarkoni-Abitbul M Tichauer D Livneh J Gershon E Zheng M Tan K John SL Slade GD Jordan J Woolf CJ Peltz G Maixner W Diatchenko L Seltzer Z Salter MW Mogil JS Genetically determined P2X7 receptor pore formation regulates variability in chronic pain sensitivity. Nat Med 2012;18:595–9. 22447075 10.1038/nm.2710 PMC3350463

[68] Tsoi LC Iyer MK Stuart PE Swindell WR Gudjonsson JE Tejasvi T Sarkar MK Li B Ding J Voorhees JJ Kang HM Nair RP Chinnaiyan AM Abecasis GR Elder JT Analysis of long non-coding RNAs highlights tissue-specific expression patterns and epigenetic profiles in normal and psoriatic skin. Genome Biol 2015;16:24. 25723451 10.1186/s13059-014-0570-4 PMC4311508

[69] Ulitsky I Bartel DP lincRNAs: genomics, evolution, and mechanisms. Cell 2013;154:26–46. 23827673 10.1016/j.cell.2013.06.020 PMC3924787

[70] Usoskin D Furlan A Islam S Abdo H Lönnerberg P Lou D Hjerling-Leffler J Haeggström J Kharchenko O Kharchenko PV Linnarsson S Ernfors P Unbiased classification of sensory neuron types by large-scale single-cell RNA sequencing. Nat Neurosci 2015;18:145–53. 25420068 10.1038/nn.3881

[71] Wahlestedt C Targeting long non-coding RNA to therapeutically upregulate gene expression. Nat Rev Drug Discov 2013;12:433–46. 23722346 10.1038/nrd4018

[72] Wang L Park HJ Dasari S Wang S Kocher JP Li W CPAT: coding-Potential Assessment Tool using an alignment-free logistic regression model. Nucleic Acids Res 2013;41:e74. 23335781 10.1093/nar/gkt006 PMC3616698

[73] Weir GA Middleton SJ Clark AJ Daniel T Khovanov N McMahon SB Bennett DL Using an engineered glutamate-gated chloride channel to silence sensory neurons and treat neuropathic pain at the source. Brain 2017;140:2570–85. 28969375 10.1093/brain/awx201 PMC5841150

[74] Wu M Huang H Chen Q Li D Zheng Z Xiong W Zhou Y Li X Zhou M Lu J Shen S Li G Leucine-rich repeat C4 protein is involved in nervous tissue development and neurite outgrowth, and induction of glioma cell differentiation. Acta Biochim Biophys Sin 2007;39:731–8. 17928921 10.1111/j.1745-7270.2007.00338.x

[75] Wu S Marie Lutz B Miao X Liang L Mo K Chang YJ Du P Soteropoulos P Tian B Kaufman AG Bekker A Hu Y Tao YX Dorsal root ganglion transcriptome analysis following peripheral nerve injury in mice. Mol Pain 2016;12:1744806916629048. 10.1177/1744806916629048 PMC4955972 27030721

[76] Xie C Yuan J Li H Li M Zhao G Bu D Zhu W Wu W Chen R Zhao Y NONCODEv4: exploring the world of long non-coding RNA genes. Nucleic Acids Res 2014;42:D98–103. 24285305 10.1093/nar/gkt1222 PMC3965073

[77] Yanai I Benjamin H Shmoish M Chalifa-Caspi V Shklar M Ophir R Bar-Even A Horn-Saban S Safran M Domany E Lancet D Shmueli O Genome-wide midrange transcription profiles reveal expression level relationships in human tissue specification. Bioinformatics 2005;21:650–9. 15388519 10.1093/bioinformatics/bti042

[78] Yarmishyn AA Batagov AO Tan JZ Sundaram GM Sampath P Kuznetsov VA Kurochkin IV HOXD-AS1 is a novel lncRNA encoded in HOXD cluster and a marker of neuroblastoma progression revealed via integrative analysis of noncoding transcriptome. BMC Genomics 2014;15(suppl 9):S7. 10.1186/1471-2164-15-S9-S7 PMC4290621 25522241

[79] Yates A Akanni W Amode MR Barrell D Billis K Carvalho-Silva D Cummins C Clapham P Fitzgerald S Gil L Girón CG Gordon L Hourlier T Hunt SE Janacek SH Johnson N Juettemann T Keenan S Lavidas I Martin FJ Maurel T McLaren W Murphy DN Nag R Nuhn M Parker A Patricio M Pignatelli M Rahtz M Riat HS Sheppard D Taylor K Thormann A Vullo A Wilder SP Zadissa A Birney E Harrow J Muffato M Perry E Ruffier M Spudich G Trevanion SJ Cunningham F Aken BL Zerbino DR Flicek P Ensembl 2016. Nucleic Acids Res 2016;44:D710–16. 26687719 10.1093/nar/gkv1157 PMC4702834

[80] Young GT Gutteridge A Fox HDE Wilbrey AL Cao L Cho LT Brown AR Benn CL Kammonen LR Friedman JH Bictash M Whiting P Bilsland JG Stevens EB Characterizing human stem cell-derived sensory neurons at the single-cell level reveals their ion channel expression and utility in pain research. Mol Ther 2014;22:1530–43. 24832007 10.1038/mt.2014.86 PMC4435594

[81] Young RS Ponting CP Identification and function of long non-coding RNAs. Essays Biochem 2013;54:113–26. 23829531 10.1042/bse0540113

[82] Zhang Y Wang J Ji LJ Li L Wei M Zhen S Wen CC Identification of key gene modules of neuropathic pain by Co-expression analysis. J Cell Biochem 2017;118:4436–43. 28460420 10.1002/jcb.26098

[83] Zhang Y Zhan Y Han N Kou Y Yin X Zhang P Analysis of temporal expression profiles after sciatic nerve injury by bioinformatic method. Sci Rep 2017;7:9818. 28852045 10.1038/s41598-017-10127-1 PMC5575162

[84] Zhao H Duan LJ Sun QL Gao YS Yang YD Tang XS Zhao DY Xiong Y Hu ZG Li CH Chen SX Liu T Yu X Identification of key pathways and genes in L4 dorsal root ganglion (DRG) after sciatic nerve injury via microarray analysis. J Invest Surg 2018:1–9. 10.1080/08941939.2018.1452996 29672183

[85] Zhao J Brown K Liem RKH Abnormal neurofilament inclusions and segregations in dorsal root ganglia of a Charcot-Marie-Tooth type 2E mouse model. PLoS One 2017;12:e0180038. 28654681 10.1371/journal.pone.0180038 PMC5487060

[86] Zhao X Tang Z Zhang H Atianjoh FE Zhao JY Liang L Wang W Guan X Kao SC Tiwari V Gao YJ Hoffman PN Cui H Li M Dong X Tao YX A long noncoding RNA contributes to neuropathic pain by silencing Kcna2 in primary afferent neurons. Nat Neurosci 2013;16:1024–31. 23792947 10.1038/nn.3438 PMC3742386

