# Supplementary material for: Comprehensive analysis of long noncoding RNA expression in dorsal root ganglion reveals cell-type specificity and dysregulation after nerve injury
Source: Pain. 2018 Oct 16;160(2):463–85. doi: 10.1097/j.pain.0000000000001416 (PMC6343954; doi:10.1097/j.pain.0000000000001416)
Supplement: SUPPLEMENTARY MATERIAL [file jop-160-463-s001.docx]

## **Supplementary material**

18 tables, 10 figures, 17 spreadsheets and Supplementary Methods are in supplementary material.

Expression data is available in PainNetworks (<http://www.painnetworks.org/>) and raw and processed sequencing data is available in GEO (super series http://www.ncbi.nlm.nih.gov/geo/query/acc.cgi?acc=GSE107182. Rodent DRG data with all supplemental material http://www.ncbi.nlm.nih.gov/geo/query/acc.cgi?acc=GSE107180, IPSc neurons data http://www.ncbi.nlm.nih.gov/geo/query/acc.cgi?acc=GSE107181).

## **Supplementary figure legends**

S. Figure 1: A,C: Ambiguously mapped reads per sample (pink bars) and read counts generated from reads mapped to multiple loci (purple bars) in mouse (A) and rat (C).# indicates excluded samples. B,D: Boxplot of Log10 Cook’s distance for all ENSEMBL genes in all samples in mouse (B) and rat (D).

S. Figure 2: Distribution of distances between TSS and novel LncRNAs (A) and TSS and annotated LncRNAs (B).

S. Figure 3: Neuron sub-type specificity of genes and LncRNAs (annotated and novel). A: PCA plot of the expression of ENSEMBL annotated genes only. Neuron sub-types are colour coded. B: Kernel density of the Tau specificity metric. The first peak of the bimodal distribution represents ubiquitous genes. The second peak represents neuron sub-type specific genes (tau > 0.8).

S. Figure 4: A: PCA plot of samples based on the expression of novel LncRNAs and ENSEMBL genes (1^st^ 10000 novel LncRNAs and ENSEMBL genes, ranked by their SD) B, C: Correlation of expression vs distance for ENSEMBL annotated (A) and novel LncRNAs (B) in IPSC and IPSC derived sensory neurons.

S. Figure 5: Over-representation analysis of GO biological process (BP) terms based on the significantly DE ENSEMBL genes (p.value < 0.05) SNT vs SHAM using the weighted Fisher exact test and the weighted KS test. GO sub-graph leading to the top 5 significantly enriched terms in Wistar rat.

S. Figure 6: Correlation of expression vs distance for ENSEMBL annotated (A) and novel LncRNAs (B) in rat DRG.

S. Figure 7: Over-representation analysis of GO biological process (BP) terms based on the significantly DE ENSEMBL genes (p.value < 0.05) SNI vs SHAM using the weighted Fisher exact test and the weighted KS test. GO sub-graph leading to the top 5 significantly enriched terms in BALB/c mice (A) and B10.D2 mice (B).

S.Figure 8: A: Soft threshold pick for WGCNA network. Topology was scaled-free with a soft threshold of 5 (A), bi-correlation matrix was raised in the power of 5 before clustering. B: Hierarchical clustering of un-merged modules, merging threshold of 0.2 is plotted with a red line. C: Gene dendrogram and module identification using dynamic tree followed by similar module clustering.

S. Figure 9: Correlation of expression vs distance for ENSEMBL annotated (A) and novel LncRNAs (B) in mouse DRG.

S. Figure 10: Gene models and primer binding location of validated LncRNAs. For every panel, track “Refseq genes” has RefSeq annotations for the respective genomic coordinates. A: LncRNA2754. B: LncRNA1528. C: LncRNA1779. D: LncRNA1291. E: LncRNA4834. F: LncRNA4714. G: LncRNA561.

## **Supplementary tables legends**

S. Table 1: RNA-sequencing quality metrics for rat DRG. GC content and percentage, insert size, Q score (Sanger qualities).

S. Table 2: Overview of the experimental design and number of uniquely mapped reads and genome coverage per sample for rat DRG.

S. Table 3: RNA-sequencing quality metrics for mouse DRG. GC content and percentage, insert size, Q score (Sanger qualities).

S. Table 4: Overview of the experimental design and number of uniquely mapped reads and genome coverage per sample for mouse DRG.

S. Table 5: Novel LncRNAs antisense of pain genes in mouse DRG.

S. Table 6: Novel LncRNAs antisense of pain genes in rat DRG.

S. Table 7: Novel intergenic LncRNAs (LincRNAs) with a pain gene as its closest genomic neighbour in mouse DRG. Distance between the LincRNA and the pain gene is given in genomic bases. Positive when downstream, negative when upstream.

S. Table 8: Novel intergenic LncRNAs (LincRNAs) with a pain gene as its closest genomic neighbour in rat DRG. Distance between the LincRNA and the pain gene is given in genomic bases. Positive when downstream, negative when upstream.

S. Table 9: Novel LncRNAs antisense of voltage-gated ion channels in mouse DRG.

S. Table 10: Novel LncRNAs antisense of voltage-gated ion channels in rat DRG.

S. Table 11: DE LncRNAs antisense of DE pain genes with opposite Log2 fold changes in IPSC vs IPSC derived sensory neurons. Log2 fold changes and p.values are for the comparison Neurons (AD2 parental line) vs IPSC AD2.

S. Table 12: Expression changes of HAGLR LncRNA in mouse SNI vs Sham and human IPSC vs IPSC-derived sensory neurons.

S. Table 13: DE LncRNAs antisense of DE pain genes with opposite Log2 fold changes in rat DRG SNT vs Sham.

S. Table 14: Intergenic LncRNAs DE in rat DRG SNT vs Sham with a pain genes as their closest genomic neighbour and a highly correlated expression. Pearson’s R correlation coefficient has been calculated on regularised log2 transform counts.

S. Table 15: Antisense LncRNAs that were significantly DE on the opposite strand of a significantly DE gene in BALB/c and B10.D2 mouse strain DRG SNI vs Sham.

S. Table 16: LncRNAs antisense of voltage gated sodium and potassium channels that were significantly DE.

S. Table 17: Intergenic LncRNAs DE in mouse DRG SNI vs Sham with a pain genes as their closest genomic neighbour and a highly correlated expression. Pearson’s R correlation coefficient has been calculated on regularised log2 transform counts.

S. Table 18: qPCR primers used for assessing the relative expression changes of novel LncRNAs and HAGLR in mouse DRG and human IPSC.

## **Supplementary data**

S. Data 1: Novel LncRNAs in mouse and rat, in syntenically conserved regions between human, mouse and rat.

S. Data 2: Novel LncRNAs in mouse DRG antisense of orthologous genes in mouse and rat.

S. Data 3: Novel LncRNAs in rat DRG antisense of orthologous genes in mouse and rat.

S. Data 4: Neuron sub-type specificity of ENSEMBL genes and novel LncRNAs. Neuron sub-type index is 1. MHN, 2. MHN (MI, IS), 3. C-LTMR, 4. MHN (IS), 5. MHN (IS), 6. MHN, 7. MHN (NS), 8. MR, 9. MHN, 10. MR. Average log2 expression holds the average expression in the neuron sub-type where the gene or LncRNAs was higher expressed. Expression SEM hold the standard error of the mean for the average expression estimator.

S. Data 5: DE analysis results and fpkm values of novel LncRNAs for IPSC-derived sensory neurons vs IPSC. Results are DESeq2 determined for the whole gene set of ENSEMBL genes and novel LncRNAs. Experimental design is ~ cell_line*condition.

S. Data 6: Expression data for all antisense LncRNAs in IPSC-derived sensory neurons vs IPSC.

S. Data 7: Expression data for all intergenic LncRNAs in IPSC-derived sensory neurons vs IPSC.

S. Data 8: Expression data for all LncRNAs antisense of pain genes in IPSC-derived sensory neurons vs IPSC.

S. Data 9: Expression data for all intergenic LncRNAs adjacent to pain genes in IPSC-derived sensory neurons vs IPSC.

S. Data 10: Novel LncRNAs in human IPSC and mouse, in syntenically conserved regions between human, mouse and rat.

S. Data 11: Novel LncRNAs in human IPSC antisense of orthologous genes in human and mouse.

S. Data 12: DE analysis results and fpkm values of novel LncRNAs for rat DRG SNT vs Sham. Results are DESeq2 determined for the whole gene set of ENSEMBL genes and novel LncRNAs. Experimental design is ~ condition.

S. Data 13: Expression data for all antisense LncRNAs in rat DRG SNT vs Sham.

S. Data 14: Expression data for all intergenic LncRNAs rat DRG SNT vs Sham.

S. Data 15: DE analysis results and fpkm values of novel LncRNAs for BALB/c and B10.D2 mouse DRG SNI vs Sham. Results are DESeq2 determined for the whole gene set of ENSEMBL genes and novel LncRNAs. Experimental design is ~ sex + strain*condition.

S. Data 16: Expression data for all antisense LncRNAs in mouse DRG SNI vs Sham.

S. Data 17: Expression data for all intergenic LncRNAs mouse DRG SNI vs Sham.

## **Supplementary Methods**

#### Single-cell mouse DRG data

Single cell mouse DRG data has been previously used and disseminated by (Li et al., 2016). We downloaded and reanalysed data in order to re-classify neuron subtypes and then looked for neuron subtype-specific expression.

#### Tissue specificity

Tissue specificity was calculated using the tau metric (Yanai et al., 2005b):


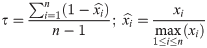


applied on regularised transformed counts of ENSEMBL genes and novel LncRNAs. R implementation:

for (i in 1:N) {

expr_est[,i] <- rowMeans(eset[,eval(parse(text=paste("colData","$",condition, sep="")))==levels(eval(parse(text=paste("colData","$",condition, sep=""))))[i]])

expr_sd[,i] <- apply(eset[,eval(parse(text=paste("colData","$",condition, sep="")))==levels(eval(parse(text=paste("colData","$",condition, sep=""))))[i]], 1, function(x) std(x))

}

max_expr <- apply(expr_est, 1, function(x) max(x))

max_norm <- sweep(expr_est, 1, max_expr, "/")

t.index <- apply(max_norm, 1, which.max)

tau <- apply(max_norm, 1, function(x) sum(1-x) / (N-1))

tau_results <- data.frame(tau = tau, t.index = t.index)

for (i in 1:nrow(tau_results)) {

tau_results$mean_expr <- max_expr

tau_results$sem[i] <- expr_sd[i, t.index[i]]

}

return(tau_results)

}

#### IPS derived human neurons

**Induced pluripotent stem cell generation**

NHDF1 (from 44-year-old female) was reprogrammed with Yamanaka retroviruses SOX2, KLF4, OCT3/4, c-MYC and NANOG (Takahashi et al., 2006), and has previously been described (Hartfield et al., 2014). AD2-01 (from 51-year-old male) (Buskin et al., in preparation) and AD4-01 (from 68-year-old male) (Melguzo et al, in preparation) were reprogrammed using the CytoTune™-iPS Reprogramming Kit (ThermoFisher). The fibroblasts to generate AD2-01 and AD4-01 were obtained from a commercial source (Lonza, CC-2511).

CytoTune-iPS reprogramming was performed as directed by the manufacturer’s instructions (ThermoFisher). The CytoTune reprogramming kit contains four Sendai virus-based reprogramming vectors each capable of expressing one of the four Yamanaka factors (KLF4, OCT3/4, SOX2 and c-MYC). Briefly, after fibroblast transduction with the four Sendai virus-based reprogramming vectors, cells are cultured for 5-6 days, with medium changes every other day (DMEM, high glucose (Sigma), 10% FBS (ThermoFisher), 1% Pen/Strep (100x, ThermoFisher), 200mM L-glutamine (Sigma), 1% non-essential amino acids (ThermoFisher)). The transduced fibroblasts are then passaged using 0.05% Trpsin-EDTA onto pre-prepared feeder layer plates containing mitotically inactivated mouse embryonic fibroblasts (MEF). 3-4 weeks after transduction, colonies should have grown to an appropriate size to allow for manual picking. Using an inverted microscope, a single colony displaying iPSC morphology is cut into 5-6 pieces using a 25 gauge needle, transferred into iPS media (KO-DMEM (ThermoFisher), 25% Knock Serum Replacement (ThermoFisher), 1% nonessential amino acids (100x, ThermoFisher), 200mM L- glutamine (Sigma), 1% Pen/Strep (100x, ThermoFisher), 8 ng/ml human FGF2 (Miltenyi Biotec)) and plated onto pre-prepared MEF plates. Colonies are allowed to attach for 48 hours, and thereafter medium changes are performed daily. iPSCs were adapted to feeder-free conditions onto Matrigel (Scientific Laboratory Supplies)-coated plates in mTeSR1 medium (ScienCell). Bulk passaging was by 0.5 mM ethylenediaminetetraacetic acid (EDTA) to make large-scale, quality-controlled stocks that were cryopreserved in liquid nitrogen. The number of feeder-free passages was kept to a minimum. When selecting iPSCs from frozen stocks for differentiation, vials with the same passage number were selected for each cell line throughout all experiments performed in this study.

The iPSC lines AD2-01 and AD4-01 were obtained through the IMI/EU sponsored StemBANCC consortium via the Human Biomaterials Resource Centre, University of Birmingham, UK (http://www.birmingham.ac.uk/facilities/hbrc).

All iPSC lines were subject to strict quality control checks before the initiation of differentiation. Quality control checks of this line included: tests for Sendai virus clearance, fluorescence-activated cell sorting (FACS) for pluripotency markers, genomic integrity checks and embryoid body tri-lineage differentiation experiments. Cells are also confirmed as negative for Mycoplasma before cryopreservation.

**Sensory neuron differentiation**

For neuronal differentiation, iPSCs were passaged onto Matrigel®-coated six-well plates using TrypLE express (ThermoFisher Scientific) and maintained in mTeSR1 supplemented with 10 μM ROCK inhibitor (ScienCell). Twenty-four hours after plating, the medium was exchanged to mouse embryonic fibroblast (MEF) conditioned medium (ScienCell) supplemented with 10 ng/ml human recombinant FGF2. Cells were allowed to expand on MEF-conditioned medium until 50% confluent, at which time differentiation was started according to Chambers et al. (2012). Briefly, medium was exchanged to knockout serum replacement (KSR) medium containing; knockout-DMEM, 15% knockout-serum replacement, 1% GlutaMAX™, 1% non-essential amino acids, 100 μM β-mercaptoethanol, 1% antibiotic/antimycotic (ThermoFisher Scientific), supplemented with the SMAD inhibitors SB431542 (Sigma, 10 μM) and LDN-193189 (Stratech, 100 nM). The medium was gradually transitioned from KSR medium to N2 medium (Neurobasal® medium, 2% B27 supplement, 1% N2 supplement, 1% GlutaMAX™, 1% antibiotic/antimycotic) (ThermoFisher Scientific) over an 11-day period. On Day 2, the small molecules CHIR99021 (Apollo Scientific, 3 μM), SU5402 (R&D Systems, 10 μM) and DAPT (Sigma, 10 μM) were also added. SMAD inhibitors were removed from the media from Day 6 onwards. On Day 11, the now immature neurons were replated onto Matrigel®-coated coverslips (25 000 cells per 13 mm coverslip) in 100% N2 medium containing human recombinant NGF, GDNF, BDNF, NT3 (all at 25 ng/ml, PeproTech) and 10 μM ROCK inhibitor. CHIR99021 (3 μM) was included in the medium until Day 14, and laminin (1 μg/ml, ThermoFisher Scientific) was supplemented into the medium from Day 20 onwards. Medium changes were performed twice weekly after replating. Cytosine β-D-arabinofuranoside (AraC, 2 μM, Sigma) was included in the medium for 24 h following replating to remove the few non-neuronal dividing cells remaining in the culture. This differentiation resulted in a completely pure neuronal culture with extensive arborized neurites by 3 weeks after the end of the small inhibitor stage.

**Ethics statement**

Human iPSC lines used in this study were derived from human skin biopsy fibroblasts, following signed informed consent. Three control cells lines were used in this study – AD2-01, AD4-01 and NHDF1. NHDF1 were reprogrammed with approval from research ethics committee: National Health Service, Health Research Authority, NRES Committee South Central, Berkshire, UK (REC 10/H0505/71).

#### Sequencing and mapping

Sequencing was performed at Oxford Genomics using the Illumina HiSeq2000 paired-end protocol with 100bp reads for rat and Illumina HiSeq4000 paired-end 100bp for mouse and 75bp for human IPSC and IPSC derived neurons. Oxford Genomics produced FastQ sequencing files which encode quality metrics following the Sanger standard, i.e. Sanger qualities, using the standard Phred score (Ewing and Green, 1998) to assess the probability that the corresponding base call is wrong. Sequencing was done in multiple sequencing lanes producing multiple technical replicates per sample. In general all these lanes gave high yield, consistent GC content, consistent and expected sequence insert between the paired-end adapters and high quality base calling. Conditions and strains were multiplexed in lanes and library batches.

Mapping to the genome was done using STAR aligner (Dobin et al., 2013). Reads were mapped on the mm10 (GRCm38) mouse genome, rn6 (Rnor_6.0) rat genome and HG38 (GRCh38) human genome, all downloaded from ENSEMBL. STAR was run suing the following parameters (according to ENCODE guidelines):

--outFilterMultimapNmax 20

max number of multiple alignments allowed for a read: if exceeded, the read is considered

unmapped

--alignSJoverhangMin 8

minimum overhang for unannotated junctions

--alignSJDBoverhangMin 1

minimum overhang for annotated junctions

--alignIntronMin 20

minimum intron length

--alignIntronMax 1000000

maximum intron length

--alignMatesGapMax 1000000

maximum genomic distance between mates

Genome was generated with --sjdbOverhang ReadLength-1.

Conditions and strains were multiplexed in lanes and library batches. Lanes were merged as BAM files after mapping (Li et al., 2009).

#### Identification of novel LncRNAs

Our analysis pipeline is illustrated in Figure 1. Workflow similar to (Cabili et al., 2011; Gerstein et al., 2014; Ilott and Ponting, 2013). (Pertea et al., 2015) is similar but we directly reconstruct novel LncRNAs on the gene level and do not identify individual transcripts of the same gene. Doing this we get a non-redundant annotation of unique genes of LncRNAs suitable for count based DE analysis (Anders et al., 2015; Love et al., 2014). The concept of islands of expression is described in (Gerstein et al., 2014). These islands of expression outside annotated genes would include all previously unknown exons of novel LncRNAs and consequently we trimmed, clustered and grouped them together into gene models. Clustering and grouping into gene models was done by taking into account identified splicing events.

Steps of the method are as follows:

1. Map reads to the genome and identify splicing junctions using STAR (Dobin et al., 2013) . Fetch ENSEMBL (Harrow et al., 2012), RefgSeq (Pruitt et al., 2014) and XenoRefSeq annotations using BiomaRt (Durinck et al., 2009) (XenoRefSeq includes all known annotated gene models from other organisms which can be accurately aligned to the genome of the organism of the experiment.). Perform operations on genomic intervals (Aboyoun et al., 2013; Carlson et al., n.d.) : Group annotations on the gene level, unlist, collapse and concatenate annotated gene models. Extend gene models by 1000bp in both ends. Identify gaps in annotations, i.e. regions not covered by known gene models.

2. Read all BAM files (mapped RNA-seq reads using STAR (Dobin et al., 2013)) of the experiment in chunks to reduce memory footprint. Consider only reads which are properly paired (Lawrence et al., 2013). In parallel (Morgan et al., 2017), using all available processors, subset read-pairs overlapping regions not belonging to known gene models. From these reads calculate genomic coverage and create coverage vectors encoded by their run-length (Pagès et al., 2017). Slice islands of these vectors above coverage threshold and having length > 100, i.e. identify islands of expression (I.o.E). These operations are executed by the custom functions “BAM_to_IOE” and “findRegs”. All operations are strand-specific.

3. Use “awk” (Aho et al., 1978) to select only the novel splicing junctions (SJ) identified by STAR. Read these SJs in R and filter out the ones identified by =< 2 reads, span < 20bp or > 100000bp. SJs are represented as the genomic coordinates of predicted introns.

4. Collapse I.o.E. Calculate the intersect of regions covered by I.o.E. in all samples. Identify I.o.E. contained in annotated introns, grouped in the gene level. Identify I.o.E. overlapped by novel SJs and novel SJs overlapping I.o.E.

A. For I.o.E. not overlapped by SJs, filter out the ones contained within introns, not belonging in the intersect of I.o.E in all samples and have a width =< 200bp. Calculate their length normalised coverage by pooling reads from all samples and select the ones that are significantly expressed at an one-sided Pr (>|Z|) < 0.1.

Feed coverage vectors in a smoothed z-score thresholding signal processing algorithm (van Brakel, 2014) implemented in the custom function “dropDetect”. Rolling coverage was calculated over a smoothing window of 31bp, the minimum coverage drop threshold was set to 5 and the minimum intron length to 20bp. Trim ends by identifying sudden drops in coverage and identify introns not detected by the aligner. Remove identified introns from the I.o.E. to reconstruct gene models.

B. Collapse SJ and calculate a disjoined set of their genomic intervals, i.e. not overlapping subset keeping track of the original regions. Calculate a mapping of co-overlapping introns (SJs) and create a GrangesList (Aboyoun et al., 2013) which groups together co-overlapping SJs. On the top level of the list are sets of co-overlapping SJs.

C. Collapse I.o.E. overlapped by SJs. Keep track of overlaps by creating a network connectivity matrix that holds information about which I.o.E. are connected to each other. Create a GrangesList (Aboyoun et al., 2013) which has at the top level sets of co-overlapping I.o.E, represented as interconnected nodes. Find overlaps between these grouped I.o.E and grouped SJs. Update the connectivity matrix to keep track of collapsed I.o.E. grouped together by SJs.

D. Within each set of grouped SJs (set of interconnected nodes), calculate overlaps between the disjoined segments and the original SJs. In this way each original SJ votes for a disjoined segment. Within each group select putative intronic areas (i.e. disjoined SJs) overlapped by > *round(top(counts) – 2*sd(counts))*. In this way we identify consensus introns (SJs) within each group.

Groups of putative introns (SJs) and putative exons (I.o.E.) are stored in parallel lists with the same top levels, each represented as a GrangesList grouped together by the connectivity matrix. Subtract introns from I.o.E., trim the edges and reconstruct gene models in a customised annotation. Discard gene models with length < 200bp. Scan the annotation for overlaps between gene models and create a non-reduntant annotation suitable for counting features at the gene level. Export the customised annotation in the form of a gene transfer format (GTF) file.

5. Count features using HTSeq (Anders et al., 2015) and the intersection not empty strategy.

6. Read HTSeq’s output and create a table of counts across all samples (TOC). Remove gene models of putative LncRNAs not expressed in at least all samples of a condition or strain. Remove putative novel LncRNAs below an average expression cut-off threshold of > 0.5 fpkm for at least one condition.

7. Fetch the genomic sequences of the expressed gene models in FASTA format and calculate coding potential using CPAT (Wang et al., 2013). Discard gene models with positive coding potential and create a set of putative LncRNAs

8. Annotate LncRNAs according to their genomic context using BedTools (Quinlan and Hall, 2010). Discard models with retained introns or intronic LncRNAs not supported by evidence of novel SJs.

9. Count features of the full gene set of annotated genes and novel LncRNAs and continue with differential expression analysis.

The R script “identify_LncRNAs.R” in http://github.com/gbaskozos/Scripts_LncRNAs carries out the identification of novel genes based on RNA-seq coverage.

The custom function BAM_to_IOE <- function(bamfile, PATH, PATH_results, igRangesExt, param, len=100, dep=2, suffix=11) takes as input a BAM file list, genomic ranges outside gene models, length and depth of continuous coverage and identifies I.o.E. The custom function dropDetect <- function(coverage, start, seqnames, strand, lag,threshold, length, influence, intron_identification) uses a smoothed z-score thresholding algorithm, adapted from (van Brakel, 2014), to identify coverage drops and peaks. It takes as input a coverage vector, start of genomic ranges, strand, the smoothing window (lag), z threshold, minimum length of intron, influence of previous peaks/drops on current signal and intron identification TRUE or FALSE and identifies transcription ends and/or introns.

We only included putative LncRNAs in this novel annotation only if they were present in all replicates of the experiment.

The pipeline was scripted in R using bioconductor (Gentleman et al., 2004) packages and custom scripts. Other R packages used in identification of LncRNAs are: data.table (Dowle et al., 2015), rtracklayer (Lawrence et al., 2009), biovizBase (Yin et al., 2017), annotationDbi (Pagès et al., 2017), IRanges (Lawrence et al., 2013).

In ribozero libraries novel intronic gene models, fully contained in the intron of a gene model and covered >= 80% of its respective introns were considered retained introns coming from sequencing of non mature transcripts and were discarded.

#### Transcription Start Sites mapping to mm10

TSS data was downloaded from FANTOM 5 database (“FANTOM5 CAGE profiles of human and mouse samples | Scientific Data,” n.d.; Lizio et al., 2015). We downloaded TSS data that has been classified as “True TSS” by the “TSS classifier”. Then we used the UCSC LiftOver tool (Meyer et al., 2012) to translate genomic coordinates from the mm9 genome to the mm10. 51% of the true TSS were unambiguously mapped to mm10.

#### Differential expression analysis

DE analysis was done in R using DESeq2 (Love et al., 2014). PCA was always performed on regularized log transformed counts (Love et al., 2014) using the top 10000 (mouse and human) or 5000 (rat)genes and novel LncRNAs ranked by observed variance. Hierarchical clustering was always performed on regularized log transformed counts using euclidean distances and complete linkage.

ENSEMBL gene annotations used: GRCh38.88 (HG38), GRCm38.87 (mm10) and Rnor_6.0.90 (rn6). To select all the annotated LncRNAs we programmatically downloaded from biomaRt all gene descriptions and gene biotypes, and then selected all genes with biotype “lincRNA”, “antisense”, “antisense RNA” “sense intronic” which denote either long intergenic non-coding RNAs, antisense long non-coding RNAs and intronic LncRNAs.

GO enrichment for DE ENSEMBL annotated genes was carried out using custom R scripts and methods developed in the topGO (Alexa and Rahnenfuhrer, 2010) R package. In the case of annotated genes we used as a background the total population of expressed genes and DE p.value < 0.05 as significance cut-off. We used the *elim* method introduced in (Alexa et al., 2006a) to compute the significance of a node dependent on the significance of its children. We tested enrichments using the Kolmogorov-Smirnov test (Alexa et al., 2006b; Alexa and Rahnenfuhrer, 2010).

Gene set enrichment analysis and GO enrichment of genes belonging to WGCNA network modules was carried out using the Fisher’s Exact test and the hypergeometric distribution and calculated by custom R scripts and the GSEA (Morgan et al., 2017) and GO stats (Falcon and Gentleman, 2007) packages. Significance cut off was p.value < 0.05 and the minimum size of a GO BP term was 100 genes.

### DE and counting features

DE analysis was done using DESeq2 (Love et al., 2014) using default settings. Significance cut-off in all cases was FDR < 0.05. Counting of features was done using HTSeq (Anders et al., 2015) and the intersection not empty strategy to resolve ambiguously counted reads.

The generalised linear models fitted for each experiment were: mouse DRG: ~ sex + strain*condition, rat DRG: ~ condition, human IPSC: ~ cell_line*condition.

#### Supplementary Bibliography

Aboyoun, P., Pages, H., Lawrence, M., 2013. GenomicRanges: Representation and manipulation of genomic intervals.

Aho, A.V., Kernighan, B.W., Weinberger, P.J., 1978. Awk – A Pattern Scanning and Processing Language (Second Edition).

Anders, S., Pyl, P.T., Huber, W., 2015. HTSeq—a Python framework to work with high-throughput sequencing data. Bioinformatics 31, 166–169. https://doi.org/10.1093/bioinformatics/btu638

Carlson, M., Pages, H., Aboyoun, P., Falcon, S., Morgan, M., Sarkar, D., Lawrence, M., n.d. GenomicFeatures: Tools for making and manipulating transcript centric annotations.

Dobin, A., Davis, C.A., Schlesinger, F., Drenkow, J., Zaleski, C., Jha, S., Batut, P., Chaisson, M., Gingeras, T.R., 2013. STAR: ultrafast universal RNA-seq aligner. Bioinformatics 29, 15–21. https://doi.org/10.1093/bioinformatics/bts635

Dowle, M., Srinivasan, A., Short, T., Saporta, S.L. with contributions from R., Antonyan, E., 2015. data.table: Extension of Data.frame.

Durinck, S., Spellman, P.T., Birney, E., Huber, W., 2009. Mapping Identifiers for the Integration of Genomic Datasets with the R/Bioconductor package biomaRt. Nat. Protoc. 4, 1184–1191. https://doi.org/10.1038/nprot.2009.97

Falcon, S., Gentleman, R., 2007. Using GOstats to test gene lists for GO term association. Bioinformatics 23, 257–8.

Lawrence, M., Gentleman, R., Carey, V., 2009. rtracklayer: an R package for interfacing with genome browsers. Bioinformatics 25, 1841–1842. https://doi.org/10.1093/bioinformatics/btp328

Lawrence, M., Huber, W., Pagès, H., Aboyoun, P., Carlson, M., Gentleman, R., Morgan, M., Carey, V., 2013. Software for Computing and Annotating Genomic Ranges. PLoS Comput. Biol. 9. https://doi.org/10.1371/journal.pcbi.1003118

Morgan, M., Obenchain, V., Lang, M., Thompson, R., 2017a. BiocParallel: Bioconductor facilities for parallel evaluation.

Morgan, M., Pagès, H., Obenchain, V., Hayden, N., 2017b. Rsamtools: Binary alignment (BAM), FASTA, variant call (BCF), and tabix file import.

Morgan, M., Falcon, S., Gentleman, R., 2017. GSEABase: Gene set enrichment data structures and methods.

Pagès, H., Carlson, M., Falcon, S., Li, N., 2017. AnnotationDbi: Annotation Database Interface.

Pagès, H., Lawrence, M., Aboyoun, P., 2017. S4Vectors: S4 implementation of vector-like and list-like objects.

Quinlan, A.R., Hall, I.M., 2010. BEDTools: a flexible suite of utilities for comparing genomic features. Bioinformatics 26, 841–842. https://doi.org/10.1093/bioinformatics/btq033

van Brakel, J.-P., 2014. algorithm - Peak signal detection in realtime timeseries data - Stack Overflow [WWW Document]. URL https://stackoverflow.com/questions/22583391/peak-signal-detection-in-realtime-timeseries-data/22640362 (accessed 5.1.18).

Wang, L., Park, H.J., Dasari, S., Wang, S., Kocher, J.-P., Li, W., 2013. CPAT: Coding-Potential Assessment Tool using an alignment-free logistic regression model. Nucleic Acids Res. 41, e74. https://doi.org/10.1093/nar/gkt006

Yin, T., Lawrence, M., Cook, D., 2017. biovizBase: Basic graphic utilities for visualization of genomic data.
