# Supplementary material for: Comprehensive analysis of long noncoding RNA expression in dorsal root ganglion reveals cell-type specificity and dysregulation after nerve injury
Source: Pain. 2018 Oct 16;160(2):463–85. doi: 10.1097/j.pain.0000000000001416 (PMC6343954; doi:10.1097/j.pain.0000000000001416)
Supplement: SUPPLEMENTARY MATERIAL [file jop-160-463-s003.doc]

| Rat RNA-seq experiment | | | | | | | | | | |
| --- | --- | --- | --- | --- | --- | --- | --- | --- | --- | --- |
| Lane | % GC | % GCmapped | σpos(%GC) | insert ± MAD | % exonic | % exon cov'ge | maxpos %N | %lowQ | %lowQend | avgQ |
| 3.1 | 51.0 ± 10.7 | 50.7 ± 10.0 | 3.97 | 151 ± 42 | 20.4 | 89.9 | 0.1 | 0 | 0 | 35.1 |
| 3.2 | 50.9 ± 11.0 | 50.5 ± 10.6 | 2.7 | 150 ± 42 | 20.5 | 89.9 | 0 | 0 | 0 | 34.5 |
|  |  |  |  |  |  |  |  |  |  |  |
| 4.1 | 53.1 ± 11.1 | 52.8 ± 10.6 | 3.52 | 153 ± 42 | 18.5 | 89 | 0.1 | 0 | 0 | 35 |
| 4.2 | 52.9 ± 11.4 | 52.5 ± 11.0 | 2.41 | 153 ± 42 | 18.5 | 89.3 | 0 | 0 | 0 | 34.5 |
|  |  |  |  |  |  |  |  |  |  |  |
| 1.1 | 50.7 ± 10.7 | 50.6 ± 10.3 | 4.3 | 154 ± 42 | 19.9 | 89.7 | 0.2 | 0 | 0 | 34.9 |
| 1.2 | 50.7 ± 11.1 | 50.4 ± 10.9 | 2.84 | 152 ± 42 | 20 | 89.8 | 0.1 | 0 | 0 | 33.3 |
|  |  |  |  |  |  |  |  |  |  |  |
| 2.1 | 51.1 ± 10.4 | 51.0 ± 10.0 | 3.74 | 158 ± 46 | 23.3 | 90.1 | 0.3 | 0 | 0 | 34.8 |
| 2.2 | 51.3 ± 10.8 | 51.0 ± 10.5 | 2.81 | 156 ± 44 | 23.4 | 90.3 | 0 | 0 | 0 | 32.8 |
