# Supplementary material for: Comprehensive analysis of long noncoding RNA expression in dorsal root ganglion reveals cell-type specificity and dysregulation after nerve injury
Source: Pain. 2018 Oct 16;160(2):463–85. doi: 10.1097/j.pain.0000000000001416 (PMC6343954; doi:10.1097/j.pain.0000000000001416)
Supplement: SUPPLEMENTARY MATERIAL [file jop-160-463-s004.doc]

| Rat RNA-seq depth | | | | |
| --- | --- | --- | --- | --- |
|  | condition | Uniquely mapped reads | Pairs of properly paired reads | Coverage based on uniquely mapped reads |
| 1 | SHAM | 120422768 | 35315197 | 9.36 |
| 2 | SHAM | 139555954 | 41749413 | 10.84 |
| 3 | SHAM | 109587232 | 34360632 | 8.51 |
| 4 | SHAM | 103491410 | 33174806 | 8.04 |
| 5 | D21_SNT | 119119050 | 32544853 | 9.26 |
| 6 | D21_SNT | 135313916 | 41347957 | 10.52 |
| 7 | D21_SNT | 116232532 | 74032392 | 9.03 |
| 8 | D21_SNT | 119358556 | 35772291 | 9.28 |
