# Supplementary material for: Comprehensive analysis of long noncoding RNA expression in dorsal root ganglion reveals cell-type specificity and dysregulation after nerve injury
Source: Pain. 2018 Oct 16;160(2):463–85. doi: 10.1097/j.pain.0000000000001416 (PMC6343954; doi:10.1097/j.pain.0000000000001416)
Supplement: SUPPLEMENTARY MATERIAL [file jop-160-463-s005.doc]

| Mouse RNA-seq experiment | | | | | | | | | | |
| --- | --- | --- | --- | --- | --- | --- | --- | --- | --- | --- |
| Lane | % GC | % GCmapped | σpos(%GC) | insert ± MAD | % exonic | % exon cov'ge | maxpos %N | %lowQ | %lowQend | avgQ |
| 1.1 | 48.3 ± 9.6 | 47.8 ± 9.4 | 4.45 | 197 ± 59 | 20.8 | 59.9 | 0.3 | 0 | 0 | 34 |
| 1.2 | 48.3 ± 10.3 | 47.8 ± 10.0 | 2.61 | 195 ± 59 | 21.8 | 61.6 | 0.6 | 0 | 0 | 31 |
|  |  |  |  |  |  |  |  |  |  |  |
| 2.1 | 48.1 ± 9.5 | 47.7 ± 9.4 | 4.46 | 191 ± 54 | 20.7 | 58.6 | 1.1 | 0 | 0.2 | 33.9 |
| 2.2 | 48.2 ± 10.2 | 47.6 ± 10.0 | 2.62 | 190 ± 53 | 21.7 | 60.3 | 1 | 0 | 0.1 | 30.8 |
|  |  |  |  |  |  |  |  |  |  |  |
| 3.1 | 48.2 ± 9.6 | 47.7 ± 9.4 | 4.45 | 192 ± 54 | 20.7 | 58.7 | 1 | 0 | 0.2 | 33.9 |
| 3.2 | 48.2 ± 10.3 | 47.6 ± 10.0 | 2.62 | 190 ± 54 | 21.7 | 60.4 | 1.1 | 0 | 0.1 | 30.9 |
|  |  |  |  |  |  |  |  |  |  |  |
| 4.1 | 48.2 ± 9.6 | 47.7 ± 9.4 | 4.45 | 192 ± 55 | 20.7 | 58.8 | 2.4 | 0 | 0.1 | 33.9 |
| 4.2 | 48.2 ± 10.3 | 47.7 ± 10.0 | 2.61 | 191 ± 55 | 21.6 | 60.5 | 1.3 | 0 | 0.1 | 30.7 |
|  |  |  |  |  |  |  |  |  |  |  |
| 5.1 | 48.1 ± 9.6 | 47.7 ± 9.4 | 4.46 | 192 ± 55 | 20.7 | 58.4 | 0.4 | 0 | 0 | 32.5 |
| 5.2 | 48.2 ± 10.3 | 47.5 ± 10.0 | 2.58 | 191 ± 55 | 21.6 | 59.9 | 0.1 | 0 | 0 | 30 |
