# Supplementary material for: Comprehensive analysis of long noncoding RNA expression in dorsal root ganglion reveals cell-type specificity and dysregulation after nerve injury
Source: Pain. 2018 Oct 16;160(2):463–85. doi: 10.1097/j.pain.0000000000001416 (PMC6343954; doi:10.1097/j.pain.0000000000001416)
Supplement: SUPPLEMENTARY MATERIAL [file jop-160-463-s006.doc]

| Mouse RNA-seq depth | | | | | | | |
| --- | --- | --- | --- | --- | --- | --- | --- |
|  | strain | sex | condition | group | Uniquely mapped reads | Pairs of properly paired reads | Coverage based on uniquely mapped reads |
| 1 | BALB.c | M | SHAM | BALB.c_SHAM | 128150964 | 58253817 | 7.25 |
| 2 | BALB.c | M | SHAM | BALB.c_SHAM | 157996400 | 70905687 | 8.83 |
| 3 | BALB.c | F | SHAM | BALB.c_SHAM | 153996898 | 71094756 | 8.70 |
| 4 | BALB.c | F | SHAM | BALB.c_SHAM | 141881266 | 62703319 | 8.02 |
| 5 | BALB.c | F | SHAM | BALB.c_SHAM | 133222250 | 60118606 | 7.54 |
| 6 | B10.D2 | M | SHAM | B10.D2_SHAM | 129686932 | 59135611 | 7.33 |
| 7 | B10.D2 | M | SHAM | B10.D2_SHAM | 131655420 | 59573126 | 7.44 |
| 8 | B10.D2 | F | SHAM | B10.D2_SHAM | 143948956 | 65180670 | 8.14 |
| 9 | BALB.c | M | SHAM | BALB.c_SHAM | 137103922 | 62141859 | 7.75 |
| 10 | B10.D2 | F | SHAM | B10.D2_SHAM | 148176972 | 67081783 | 8.37 |
| 11 | B10.D2 | M | SHAM | B10.D2_SHAM | 155847954 | 71180013 | 8.81 |
| 12 | BALB.c | M | SNI | BALB.c_SNI | 116681670 | 53381176 | 6.60 |
| 13 | BALB.c | F | SNI | BALB.c_SNI | 152372720 | 69878368 | 8.61 |
| 14 | BALB.c | F | SNI | BALB.c_SNI | 149408206 | 67104230 | 8.45 |
| 15 | B10.D2 | F | SNI | B10.D2_SNI | 140526254 | 63262815 | 7.94 |
| 16 | BALB.c | M | SNI | BALB.c_SNI | 135574098 | 61279835 | 7.66 |
| 17 | B10.D2 | F | SNI | B10.D2_SNI | 153651302 | 69507207 | 8.69 |
| 18 | B10.D2 | F | SNI | B10.D2_SNI | 158587850 | 72285022 | 8.97 |
| 19 | B10.D2 | M | SNI | B10.D2_SNI | 146210692 | 65741153 | 8.27 |
| 20 | B10.D2 | M | SNI | B10.D2_SNI | 138142970 | 62903003 | 7.81 |
