# Supplementary material for: Comprehensive analysis of long noncoding RNA expression in dorsal root ganglion reveals cell-type specificity and dysregulation after nerve injury
Source: Pain. 2018 Oct 16;160(2):463–85. doi: 10.1097/j.pain.0000000000001416 (PMC6343954; doi:10.1097/j.pain.0000000000001416)
Supplement: SUPPLEMENTARY MATERIAL [file jop-160-463-s007.doc]

| Mouse DRG novel LncRNAs antisense of pain genes | | | |
| --- | --- | --- | --- |
| LncRNA name (coordinates) | LncRNA ID | Pain gene ENSEMBL ID | Pain Gene symbol |
| 11:55394500-55395410(+) | LncRNA2153 | ENSMUSG00000018593 | Sparc |
| 11:70240212-70242700(+) | LncRNA2170 | ENSMUSG00000000320 | Alox12 |
| 11:73297432-73307708(-) | LncRNA2276 | ENSMUSG00000043029 | Trpv3 |
| 12:113144963-113145499(-) | LncRNA2486 | ENSMUSG00000006356 | Crip2 |
| 14:63141461-63144107(-) | LncRNA2750 | ENSMUSG00000021939 | Ctsb |
| 16:35299876-35310125(-) | LncRNA3043 | ENSMUSG00000022840 | Adcy5 |
| 2:131936420-131936829(-) | LncRNA467 | ENSMUSG00000079037 | Prnp |
| 5:43868670-43869133(-) | LncRNA1094 | ENSMUSG00000029084 | Cd38 |
| 6:92158445-92168511(-) | LncRNA5190 | ENSMUSG00000005893 | Nr2c2 |
| 6:118167896-118169229(+) | LncRNA5075 | ENSMUSG00000030110 | Ret |
| 7:114631428-114631907(+) | LncRNA1441 | ENSMUSG00000030669 | Calca |
| 7:114635603-114715554(+) | LncRNA1442 | ENSMUSG00000030669 | Calca |
| 9:119730828-119763742(+) | LncRNA5774 | ENSMUSG00000034115 | Scn11a |
