# Supplementary material for: Comprehensive analysis of long noncoding RNA expression in dorsal root ganglion reveals cell-type specificity and dysregulation after nerve injury
Source: Pain. 2018 Oct 16;160(2):463–85. doi: 10.1097/j.pain.0000000000001416 (PMC6343954; doi:10.1097/j.pain.0000000000001416)
Supplement: SUPPLEMENTARY MATERIAL [file jop-160-463-s008.doc]

| Rat DRG novel LncRNAs antisense of pain genes | | | |
| --- | --- | --- | --- |
| LncRNA name (coordinates) | LncRNA ID | Pain gene ENSEMBL ID | Pain Gene symbol |
| 1:36353933-36354161(-) | LncRNA623 | ENSRNOG00000017601 | Srd5a1 |
| 1:84239119-84313048(-) | LncRNA711 | ENSRNOG00000018369 | Prx |
| 1:84321378-84322196(-) | LncRNA712 | ENSRNOG00000018369 | Prx |
| 1:184188353-184188948(+) | LncRNA364 | ENSRNOG00000011130 | Calca |
| 1:237907633-237910990(+) | LncRNA466 | ENSRNOG00000017469 | Anxa1 |
| 2:44293038-44314681(-) | LncRNA1595 | ENSRNOG00000013963 | Il6st |
| 2:187160101-187160384(+) | LncRNA6942 | ENSRNOG00000013953 | Ntrk1 |
| 2:193900841-193901227(-) | LncRNA1853 | ENSRNOG00000023226 | S100a10 |
| 4:9610766-9626799(-) | LncRNA2925 | ENSRNOG00000021441 | Reln |
| 4:9627458-9634043(-) | LncRNA2926 | ENSRNOG00000021441 | Reln |
| 4:157375967-157495368(+) | LncRNA2856 | ENSRNOG00000016294 | Cd4 |
| 5:3737558-3783928(-) | LncRNA3507 | ENSRNOG00000007354 | Trpa1 |
| 6:103616022-103687750(+) | LncRNA8506 | ENSRNOG00000006599 | NA |
| 7:41475712-41475995(-) | LncRNA4642 | ENSRNOG00000023896 | Dusp6 |
| 7:126680098-126683214(-) | LncRNA4757 | ENSRNOG00000021463 | Ppara |
| 8:58755493-58755777(+) | LncRNA4864 | ENSRNOG00000000196 | Cyp19a1 |
| 8:75723255-75723599(-) | LncRNA5114 | ENSRNOG00000010362 | Anxa2 |
| 8:95968358-95969347(-) | LncRNA5148 | ENSRNOG00000011071 | Nt5e |
| 8:96004232-96017662(-) | LncRNA5149 | ENSRNOG00000011071 | Nt5e |
