# Supplementary material for: Comprehensive analysis of long noncoding RNA expression in dorsal root ganglion reveals cell-type specificity and dysregulation after nerve injury
Source: Pain. 2018 Oct 16;160(2):463–85. doi: 10.1097/j.pain.0000000000001416 (PMC6343954; doi:10.1097/j.pain.0000000000001416)
Supplement: SUPPLEMENTARY MATERIAL [file jop-160-463-s009.doc]

| Novel intergenic LncRNAs in mouse DRG with a pain gene as their closest genomic neighbour | | | | |
| --- | --- | --- | --- | --- |
| LncRNA name (coordinates) | LncRNA ID | Pain gene ENSEMBL ID | Pain Gene symbol | Distance |
| 11:63130153-63186753(+) | LncRNA2166 | ENSMUSG00000018217 | Pmp22 | -1018 |
| 11:73310429-73310720(+) | LncRNA2172 | ENSMUSG00000043029 | Trpv3 | -10066 |
| 11:97107759-97174736(-) | LncRNA6282 | ENSMUSG00000001441 | Npepps | -31106 |
| 12:104438172-104438855(+) | LncRNA2411 | ENSMUSG00000021091 | Serpina3n | -23843 |
| 13:55461928-55463245(+) | LncRNA6551 | ENSMUSG00000074886 | Grk6 | -1001 |
| 15:72507982-72510044(-) | LncRNA7041 | ENSMUSG00000036760 | Kcnk9 | -2075 |
| 16:93008313-93008946(-) | LncRNA3084 | ENSMUSG00000022952 | Runx1 | 182164 |
| 17:13019120-13032557(+) | LncRNA7242 | ENSMUSG00000006818 | Sod2 | -1001 |
| 19:16389521-16404555(+) | LncRNA3423 | ENSMUSG00000024639 | Gnaq | -2058 |
| 19:22075141-22084363(+) | LncRNA3427 | ENSMUSG00000052387 | Trpm3 | 54756 |
| 19:22110149-22121663(+) | LncRNA3428 | ENSMUSG00000052387 | Trpm3 | 17456 |
| 19:59238371-59246400(+) | LncRNA3468 | ENSMUSG00000040901 | Kcnk18 | -1001 |
| 19:60213784-60241092(-) | LncRNA7691 | ENSMUSG00000045052 | Prlhr | -225641 |
| 19:61096985-61110801(+) | LncRNA3471 | ENSMUSG00000003228 | Grk5 | -9619 |
| 2:148398192-148412506(+) | LncRNA340 | ENSMUSG00000037014 | Sstr4 | -13691 |
| 4:132078749-132109721(-) | LncRNA4714 | ENSMUSG00000050511 | Oprd1 | -1005 |
| 5:35200106-35208079(+) | LncRNA970 | ENSMUSG00000045318 | Adra2c | 67197 |
| 6:99652802-99691088(-) | LncRNA5199 | ENSMUSG00000030069 | Prok2 | -20211 |
| 6:126602358-126635461(-) | LncRNA1339 | ENSMUSG00000047976 | Kcna1 | -4936 |
| 7:100985647-100995563(-) | LncRNA5452 | ENSMUSG00000032860 | P2ry2 | -1005 |
| 9:81600537-81627190(-) | LncRNA1916 | ENSMUSG00000049511 | Htr1b | -25730 |
| 9:119598332-119607455(-) | LncRNA1947 | ENSMUSG00000034533 | Scn10a | -1001 |
| 9:119740632-119746657(-) | LncRNA1950 | ENSMUSG00000034115 | Scn11a | -7102 |
