# Supplementary material for: Comprehensive analysis of long noncoding RNA expression in dorsal root ganglion reveals cell-type specificity and dysregulation after nerve injury
Source: Pain. 2018 Oct 16;160(2):463–85. doi: 10.1097/j.pain.0000000000001416 (PMC6343954; doi:10.1097/j.pain.0000000000001416)
Supplement: SUPPLEMENTARY MATERIAL [file jop-160-463-s010.doc]

| Novel intergenic LncRNAs in rat DRG with a pain gene as their closest genomic neighbour | | | | |
| --- | --- | --- | --- | --- |
| LncRNA name (coordinates) | LncRNA ID | Pain gene ENSEMBL ID | Pain Gene symbol | Distance |
| LncRNA72 | 1:43357117-43357455(+) | ENSRNOG00000018191 | Oprm1 | 97348 |
| LncRNA6515 | 1:80611565-80611819(-) | ENSRNOG00000018454 | Apoe | -1076 |
| LncRNA218 | 1:104559137-104559721(+) | ENSRNOG00000014530 | Nav2 | 16868 |
| LncRNA6555 | 1:124041527-124042593(-) | ENSRNOG00000010853 | Chrna7 | 2331 |
| LncRNA254 | 1:126707509-126707906(+) | ENSRNOG00000011526 | Pcsk6 | 41602 |
| LncRNA255 | 1:126708538-126709260(+) | ENSRNOG00000011526 | Pcsk6 | 40248 |
| LncRNA6263 | 1:156532112-156551299(+) | ENSRNOG00000022635 | Dlg2 | 1029 |
| LncRNA6602 | 1:166038470-166043910(-) | ENSRNOG00000019283 | P2ry2 | 1046 |
| LncRNA6668 | 1:216972238-216972591(-) | ENSRNOG00000054917 | Mrgpre | 1055 |
| LncRNA481 | 1:239947027-239950543(+) | ENSRNOG00000027770 | Trpm3 | 404606 |
| LncRNA482 | 1:239976336-239989419(+) | ENSRNOG00000027770 | Trpm3 | 365730 |
| LncRNA483 | 1:240230190-240236136(+) | ENSRNOG00000027770 | Trpm3 | 119013 |
| LncRNA6358 | 1:240351693-240354147(+) | ENSRNOG00000027770 | Trpm3 | 1002 |
| LncRNA6911 | 2:153776661-153802312(+) | ENSRNOG00000009514 | Mme | 1037 |
| LncRNA1830 | 2:179580859-179582769(-) | ENSRNOG00000054204 | Gria2 | -1539 |
| LncRNA1845 | 2:188962007-188968672(-) | ENSRNOG00000020778 | Chrnb2 | -119898 |
| LncRNA1406 | 2:194005474-194073083(+) | ENSRNOG00000023226 | S100a10 | -104238 |
| LncRNA6959 | 2:197654221-197654597(+) | ENSRNOG00000021157 | NA | 1189 |
| LncRNA7285 | 2:240868804-240888490(-) | ENSRNOG00000023258 | Nfkb1 | 2115 |
| LncRNA7011 | 2:264042296-264114519(+) | ENSRNOG00000010325 | Ptger3 | -62598 |
| LncRNA7535 | 3:11450542-11474804(-) | ENSRNOG00000013973 | Lcn2 | 32996 |
| LncRNA2321 | 3:12776875-12878720(-) | ENSRNOG00000017019 | Lmx1b | 90006 |
| LncRNA7673 | 3:124082535-124082771(-) | ENSRNOG00000021256 | Adra1d | -46787 |
| LncRNA2203 | 3:127978865-127983805(+) | ENSRNOG00000004810 | Plcb1 | 171264 |
| LncRNA2204 | 3:128113557-128113971(+) | ENSRNOG00000004810 | Plcb1 | 41098 |
| LncRNA10015 | 3:128444601-128445050(+) | ENSRNOG00000004810 | Plcb1 | -25056 |
| LncRNA7455 | 3:128602478-128688346(+) | ENSRNOG00000033119 | Plcb4 | 68453 |
| LncRNA2494 | 3:129383147-129383590(-) | ENSRNOG00000005509 | Pak7 | 25656 |
| LncRNA2300 | 3:177223778-177224015(+) | ENSRNOG00000016768 | Oprl1 | 1722 |
| LncRNA2301 | 3:177232664-177239901(+) | ENSRNOG00000016768 | Oprl1 | -1001 |
| LncRNA3096 | 4:123860192-123866644(-) | ENSRNOG00000009019 | Slc6a6 | 146728 |
| LncRNA7857 | 4:146260088-146275790(+) | ENSRNOG00000006527 | Slc6a1 | 1072 |
| LncRNA2828 | 4:146468169-146468539(+) | ENSRNOG00000007420 | Hrh1 | -11095 |
| LncRNA3127 | 4:150249008-150249315(-) | ENSRNOG00000014751 | Ret | 4636 |
| LncRNA3172 | 4:169541564-169552161(-) | ENSRNOG00000008766 | Grin2b | -8226 |
| LncRNA3196 | 5:3754456-3761754(+) | ENSRNOG00000007354 | Trpa1 | 21493 |
| LncRNA3197 | 5:3763292-3763707(+) | ENSRNOG00000007354 | Trpa1 | 19540 |
| LncRNA3606 | 5:75546721-75556028(-) | ENSRNOG00000013656 | Lpar1 | -1014 |
| LncRNA3390 | 5:123528297-123529199(+) | ENSRNOG00000007410 | Dab1 | 375967 |
| LncRNA3391 | 5:123824591-123826090(+) | ENSRNOG00000007410 | Dab1 | 79076 |
| LncRNA3788 | 6:9686274-9690983(+) | ENSRNOG00000015603 | NA | 99439 |
| LncRNA3844 | 6:43815689-43817405(+) | ENSRNOG00000054259 | Klf11 | 12540 |
| LncRNA8727 | 6:128435212-128451897(-) | ENSRNOG00000010711 | Dicer1 | 1029 |
| LncRNA8804 | 7:41582475-41627947(+) | ENSRNOG00000023896 | Dusp6 | -103083 |
| LncRNA4847 | 8:50186956-50328931(+) | ENSRNOG00000016847 | Bace1 | -24595 |
| LncRNA5076 | 8:53966266-53983337(-) | ENSRNOG00000031890 | Ncam1 | 64908 |
| LncRNA5077 | 8:54059819-54065375(-) | ENSRNOG00000031890 | Ncam1 | 158461 |
| LncRNA4858 | 8:54990257-54990498(+) | ENSRNOG00000009848 | Il18 | 3361 |
| LncRNA5105 | 8:69165792-69169147(-) | ENSRNOG00000010176 | Map2k1 | 1034 |
| LncRNA5135 | 8:89144412-89149303(-) | ENSRNOG00000013042 | Htr1b | 13421 |
| LncRNA5136 | 8:89165979-89170397(-) | ENSRNOG00000013042 | Htr1b | 34988 |
| LncRNA5310 | 9:62102519-62106196(+) | ENSRNOG00000032659 | Plcl1 | 185209 |
| LncRNA5311 | 9:62220850-62223020(+) | ENSRNOG00000032659 | Plcl1 | 68385 |
| LncRNA9608 | 9:70788920-70796020(-) | ENSRNOG00000012961 | NA | 1007 |
| LncRNA5357 | 9:98376133-98376670(+) | ENSRNOG00000019926 | Ramp1 | -11928 |
| LncRNA5660 | X:27864020-27864435(+) | ENSRNOG00000004118 | Frmpd4 | 208391 |
| LncRNA5790 | X:107409312-107494462(+) | ENSRNOG00000002419 | Plp1 | 1610 |
