# Supplementary material for: Comprehensive analysis of long noncoding RNA expression in dorsal root ganglion reveals cell-type specificity and dysregulation after nerve injury
Source: Pain. 2018 Oct 16;160(2):463–85. doi: 10.1097/j.pain.0000000000001416 (PMC6343954; doi:10.1097/j.pain.0000000000001416)
Supplement: SUPPLEMENTARY MATERIAL [file jop-160-463-s011.doc]

| Novel LncRNAs in mouse DRG antisense of voltage gated ion channels | | | |
| --- | --- | --- | --- |
| LncRNA name (coordinates) | LncRNA ID | Sense gene ENSEMBL ID | Sense Gene symbol |
| Potassium Channels | | | |
| 1:87332176-87402632(+) | LncRNA48 | ENSMUSG00000079436 | Kcnj13 |
| 11:33844702-33880117(-) | LncRNA2252 | ENSMUSG00000020155 | Kcnmb1 |
| 15:99241659-99282134(-) | LncRNA7074 | ENSMUSG00000037579 | Kcnh3 |
| 3:32469532-32472694(+) | LncRNA522 | ENSMUSG00000091091 | Kcnmb3 |
| 3:65067032-65110222(-) | LncRNA633 | ENSMUSG00000027827 | Kcnab1 |
| 3:107111107-107111410(-) | LncRNA662 | ENSMUSG00000040724 | Kcna2 |
| 4:152391227-152391657(+) | LncRNA818 | ENSMUSG00000028931 | Kcnab2 |
| 7:143176517-143186434(-) | LncRNA1538 | ENSMUSG00000009545 | Kcnq1 |
| Sodium Channels | | | |
| 2:66665266-66676581(+) | LncRNA266 | ENSMUSG00000034810 | Scn7a |
| 9:119730828-119763742(+) | LncRNA5774 | ENSMUSG00000034115 | Scn11a |
| TRP Channels | | | |
| 11:73297432-73307708(-) | LncRNA2276 | ENSMUSG00000043029 | Trpv3 |
