# Supplementary material for: Comprehensive analysis of long noncoding RNA expression in dorsal root ganglion reveals cell-type specificity and dysregulation after nerve injury
Source: Pain. 2018 Oct 16;160(2):463–85. doi: 10.1097/j.pain.0000000000001416 (PMC6343954; doi:10.1097/j.pain.0000000000001416)
Supplement: SUPPLEMENTARY MATERIAL [file jop-160-463-s012.doc]

| Novel LncRNAs in rat DRG antisense of voltage gated ion channels | | | |
| --- | --- | --- | --- |
| LncRNA name (coordinates) | LncRNA ID | Sense gene ENSEMBL ID | Sense Gene symbol |
| Calcium channels | | | |
| 4:151402902-151409369(-) | LncRNA3129 | ENSRNOG00000008031 | Cacna2d4 |
| Potassium Channels | | | |
| 1:216375194-216380918(-) | LncRNA916 | ENSRNOG00000020532 | Kcnq1 |
| 2:209895961-209921066(-) | LncRNA1883 | ENSRNOG00000050416 | Kcna10 |
| 4:159263373-159286693(+) | LncRNA2859 | ENSRNOG00000052486 | Kcna6 |
| 6:36819126-36821854(+) | LncRNA3837 | ENSRNOG00000004899 | Kcns3 |
| Sodium Channels | | | |
| 3:52509234-52510407(+) | LncRNA2066 | ENSRNOG00000053122 | Scn1a |
| 3:52782292-52783549(+) | LncRNA7353 | ENSRNOG00000029342 | Scn7a |
| 8:49415408-49434636(-) | LncRNA9305 | ENSRNOG00000016221 | Scn2b |
| Chloride Channels | | | |
| 5:164802402-164871681(+) | LncRNA8228 | ENSRNOG00000008345 | Clcn6 |
| TRP Channels | | | |
| 5:3737558-3783928(-) | LncRNA3507 | ENSRNOG00000007354 | Trpa1 |
