# Supplementary material for: Comprehensive analysis of long noncoding RNA expression in dorsal root ganglion reveals cell-type specificity and dysregulation after nerve injury
Source: Pain. 2018 Oct 16;160(2):463–85. doi: 10.1097/j.pain.0000000000001416 (PMC6343954; doi:10.1097/j.pain.0000000000001416)
Supplement: SUPPLEMENTARY MATERIAL [file jop-160-463-s013.doc]

| DE LncRNAs antisense of DE pain genes with opposite Log2 fold changes | | | | | | | |
| --- | --- | --- | --- | --- | --- | --- | --- |
| LncRNA ID | LncRNA name (coordinates) | Sense gene ID | Gene symbol | LncRNA Log2 fold change | LncRNA adj. p.value | Gene Log2 fold change | Gene adj. p.value |
| ENSG00000234377 | RNF219-AS1 | ENSG00000136160 | EDNRB | 5.92 | < 0.001 | -3.81 | < 0.001 |
| ENSG00000261762 | NA | ENSG00000169684 | CHRNA5 | 5.35 | < 0.001 | -2.43 | < 0.001 |
| ENSG00000215067 | ALOX12-AS1 | ENSG00000108839 | ALOX12 | 1.77 | < 0.001 | -8.36 | < 0.001 |
| ENSG00000264107 | NA | ENSG00000196712 | NF1 | -2.72 | < 0.001 | 1.59 | < 0.001 |
| ENSG00000263766 | NA | ENSG00000141279 | NPEPPS | -0.99 | 0.049 | 1 | < 0.001 |
| ENSG00000204044 | NA | ENSG00000124140 | SLC12A5 | -1.49 | < 0.001 | 2.59 | < 0.001 |
| ENSG00000225756 | DBH-AS1 | ENSG00000123454 | DBH | 2.14 | < 0.001 | -5.31 | < 0.001 |
| LncRNA5804 | 8:90053955-90060834(+) | ENSG00000104327 | CALB1 | 2.94 | < 0.001 | -4.78 | < 0.001 |
