# Supplementary material for: Comprehensive analysis of long noncoding RNA expression in dorsal root ganglion reveals cell-type specificity and dysregulation after nerve injury
Source: Pain. 2018 Oct 16;160(2):463–85. doi: 10.1097/j.pain.0000000000001416 (PMC6343954; doi:10.1097/j.pain.0000000000001416)
Supplement: SUPPLEMENTARY MATERIAL [file jop-160-463-s014.doc]

| Expression changes of HAGLR LncRNA | | | | | |
| --- | --- | --- | --- | --- | --- |
| Organism and condition | Gene ID | baseMean | log2FoldChange | Adjusted p.value | symbol |
| Human neurons vs IPSc | ENSG00000224189 | 261.6 | 8.74 | < 0.001 | HAGLR |
| BALB/c mouse DRG SNI vs Sham | ENSMUSG00000075277 | 151.5 | -0.46 | 0.004 | Haglr |
| B10.D2 mouse DRG SNI vs Sham | ENSMUSG00000075277 | 151.5 | -0.49 | 0.001 | Haglr |
