# Supplementary material for: Comprehensive analysis of long noncoding RNA expression in dorsal root ganglion reveals cell-type specificity and dysregulation after nerve injury
Source: Pain. 2018 Oct 16;160(2):463–85. doi: 10.1097/j.pain.0000000000001416 (PMC6343954; doi:10.1097/j.pain.0000000000001416)
Supplement: SUPPLEMENTARY MATERIAL [file jop-160-463-s015.doc]

| DE LncRNAs antisense of DE pain genes with opposite Log2 fold changes in rat DRG | | | | | | | |
| --- | --- | --- | --- | --- | --- | --- | --- |
| LncRNA ID | LncRNA name (coordinates) | Sense gene ID | Gene symbol | LncRNA Log2 fold change | LncRNA adj. p.value | Gene Log2 fold change | Gene adj. p.value |
| LncRNA6781 | 2:26247635-26263258(+) | ENSRNOG00000025406 | Iqgap2 | -2.57 | < 0.001 | 0.54 | 0.008 |
| LncRNA7193 | 2:149445646-149481258(-) | ENSRNOG00000010680 | Med12l | 0.68 | 0.01 | -0.75 | < 0.001 |
| LncRNA1982 | 3:5991884-5997326(+) | ENSRNOG00000007681 | Brd3 | -0.97 | < 0.001 | 0.46 | 0.01 |
| LncRNA2287 | 3:171273204-171276292(+) | ENSRNOG00000006314 | Zbp1 | -3.42 | < 0.001 | 1.42 | 0.006 |
| LncRNA3645 | 5:107733448-107751000(-) | ENSRNOG00000006615 | Mtap | 1.27 | 0.04 | -0.51 | 0.005 |
| LncRNA3709 | 5:144326425-144423068(-) | ENSRNOG00000010841 | Col8a2 | -0.61 | < 0.001 | 0.43 | 0.002 |
| LncRNA4282 | 7:13751062-13755737(+) | ENSRNOG00000007509 | Slc1a6 | -1.7 | 0.002 | 1.58 | < 0.001 |
| LncRNA9091 | 7:141680351-141760307(-) | ENSRNOG00000056106 | Dip2b | -0.79 | 0.03 | 0.63 | 0.002 |
| LncRNA5107 | 8:70169463-70191926(-) | ENSRNOG00000010634 | Megf11 | -0.88 | < 0.001 | 2.16 | < 0.001 |
