# Supplementary material for: Comprehensive analysis of long noncoding RNA expression in dorsal root ganglion reveals cell-type specificity and dysregulation after nerve injury
Source: Pain. 2018 Oct 16;160(2):463–85. doi: 10.1097/j.pain.0000000000001416 (PMC6343954; doi:10.1097/j.pain.0000000000001416)
Supplement: SUPPLEMENTARY MATERIAL [file jop-160-463-s016.doc]

| Intergenic LncRNAs close and highly correlated with pain genes | | | | | | | | | |
| --- | --- | --- | --- | --- | --- | --- | --- | --- | --- |
| LncRNA ID | LncRNA name (coordinates) | Closest gene symbol | Distance | LncRNA Log2 fold change | LncRNA adj. p.value | Gene Log2 fold change | Gene adj. p.value | Correlation | Cor. Adj. p.value |
| LncRNA1830 | 2:179580859-179582769(-) | Gria2 | -1539 | -2.31 | < 0.001 | -1.89 | < 0.001 | 0.98 | 0.006 |
| LncRNA2301 | 3:177232664-177239901(+) | Oprl1 | -1001 | -0.92 | 0.002 | -0.68 | 0.009 | 0.98 | 0.006 |
| LncRNA3172 | 4:169541564-169552161(-) | Grin2b | -8226 | 1.31 | < 0.001 | 1.20 | < 0.001 | 0.98 | 0.006 |
| LncRNA3196 | 5:3754456-3761754(+) | Trpa1 | 21493 | -2.43 | < 0.001 | -0.97 | 0.03 | 0.94 | 0.02 |
| ENSRNOG00000062160 | NA | Chrnb4 | -555 | -3.58 | < 0.001 | -2.59 | 0.24 | 0.94 | 0.02 |
