# Supplementary material for: Comprehensive analysis of long noncoding RNA expression in dorsal root ganglion reveals cell-type specificity and dysregulation after nerve injury
Source: Pain. 2018 Oct 16;160(2):463–85. doi: 10.1097/j.pain.0000000000001416 (PMC6343954; doi:10.1097/j.pain.0000000000001416)
Supplement: SUPPLEMENTARY MATERIAL [file jop-160-463-s017.doc]

| **Balb/c** | | | | | | |
| --- | --- | --- | --- | --- | --- | --- |
| LncRNA ID | LncRNA symbol (coordinates) | Sense gene symbol | LncRNA Log2 Fold change | LncRNA adj. p.value | Gene Log2 Fold change | Gene adj. p.value |
| ENSMUSG00000097649 | NA | Rftn2 | 0.49 | 0.04 | 0.48 | < 0.001 |
| ENSMUSG00000104677 | NA | Nbea | -0.31 | 0.002 | 0.15 | 0.01 |
| ENSMUSG00000097596 | NA | Kcna6 | -1.00 | < 0.001 | 0.21 | 0.04 |
| ENSMUSG00000100600 | A230077H06Rik | Vstm2b | -1.83 | < 0.001 | -1.40 | < 0.001 |
| LncRNA6043 | 10:97669599-97680412(-) | Epyc | -0.75 | < 0.001 | 0.89 | 0.01 |
| LncRNA2252 | 11:33844702-33880117(-) | Kcnmb1 | -0.44 | < 0.001 | -0.33 | 0.004 |
| LncRNA2754 | 14:68087301-68089062(-) | Nefl | -0.53 | 0.002 | -0.43 | < 0.001 |
| LncRNA531 | 3:56001321-56111248(+) | Nbea | -0.24 | 0.001 | 0.15 | 0.01 |
| LncRNA4536 | 4:109395434-109413760(+) | NA | -0.33 | 0.03 | -0.56 | < 0.001 |
| LncRNA963 | 5:30880183-30935677(+) | Cgref1 | -1.2 | 0.04 | 0.24 | 0.002 |
| **B10.D2** | | | | | | |
| LncRNA ID | LncRNA symbol (coordinates) | Sense gene symbol | LncRNA Log2 Fold change | LncRNA adj. p.value | Gene Log2 Fold change | Gene adj. p.value |
| ENSMUSG00000100600 | A230077H06Rik | Vstm2b | -1.74 | < 0.001 | -2.08 | < 0.001 |
| LncRNA203 | 1:171379256-171380555(-) | Nectin4 | -0.73 | 0.02 | -0.44 | < 0.001 |
| LncRNA6043 | 10:97669599-97680412(-) | Epyc | -0.73 | < 0.001 | 1.07 | 0.001 |
| LncRNA2754 | 14:68087301-68089062(-) | Nefl | -0.49 | 0.006 | -0.47 | < 0.001 |
| LncRNA1528 | 7:130932111-130937027(-) | Htra1 | -0.45 | 0.02 | -0.23 | 0.001 |
