# Supplementary material for: Comprehensive analysis of long noncoding RNA expression in dorsal root ganglion reveals cell-type specificity and dysregulation after nerve injury
Source: Pain. 2018 Oct 16;160(2):463–85. doi: 10.1097/j.pain.0000000000001416 (PMC6343954; doi:10.1097/j.pain.0000000000001416)
Supplement: SUPPLEMENTARY MATERIAL [file jop-160-463-s018.doc]

| **LncRNAs antisense of Potassium channels** | | | | | | |
| --- | --- | --- | --- | --- | --- | --- |
| LncRNA ID | LncRNA symbol (coordinates) | Sense gene symbol | LncRNA Log2 Fold change | LncRNA adj. p.value | Gene Log2 Fold change | Gene adj. p.value |
| **BALB/c Mouse DRG SNI vs Sham** | | | | | | |
| ENSMUSG00000097596 | NA | Kcna6 | -1.00 | < 0.001 | 0.21 | 0.04 |
| LncRNA2252 | 11:33844702-33880117(-) | Kcnmb1 | -0.44 | < 0.001 | -0.33 | 0.004 |
| **B10.D2 Mouse DRG SNI vs Sham** | | | | | | |
| ENSMUSG00000097596 | NA | Kcna6 | -1.09 | < 0.001 | 0.02 | 0.9 |
| LncRNA2252 | 11:33844702-33880117(-) | Kcnmb1 | -0.36 | < 0.001 | -0.2 | 0.18 |
| **Rat DRG SNT vs Sham** | | | | | | |
| LncRNA916 | 1:216375194-216380918(-) | Kcnq1 | -0.82 | 0.03 | -0.26 | 0.68 |
| LncRNA2859 | 4:159263373-159286693(+) | Kcna6 | -2.66 | 0.01 | 0.17 | 0.64 |
| LncRNA3837 | 6:36819126-36821854(+) | Kcns3 | -1.57 | 0.01 | -2.66 | < 0.001 |
| **Human IPSC-derived neurons vs IPSC** | | | | | | |
| ENSG00000226009 | KCNIP2-AS1 | KCNIP2 | -1.7 | 0.009 | 3 | < 0.001 |
| ENSG00000269821 | KCNQ1OT1 | KCNQ1 | 1.39 | < 0.001 | -4.56 | < 0.001 |
| ENSG00000267365 | KCNJ2-AS1 | KCNJ2 | -0.99 | 0.03 | 0.28 | 0.3 |
| ENSG00000244558 | NA | KCNK15 | 4.32 | < 0.001 | -0.11 | 0.8 |
| ENSG00000177410 | ZFAS1 | KCNB1 | -1.42 | < 0.001 | 5.8 | < 0.001 |
| LncRNA2321 | 10:77248763-77249835(+) | KCNMA1 | 7.35 | < 0.001 | 4.25 | < 0.001 |
| LncRNA1584 | 6:72614790-72622130(-) | KCNQ1 | 4.05 | < 0.001 | 2.82 | < 0.001 |
| **LncRNAs antisense of Sodium channels** | | | | | | |
| LncRNA ID | LncRNA symbol (coordinates) | Sense gene symbol | LncRNA Log2 Fold change | LncRNA adj. p.value | Gene Log2 Fold change | Gene adj. p.value |
| **BALB/c Mouse DRG SNI vs Sham** | | | | | | |
| ENSMUSG00000087301 | NA | Scn9a | -1.11 | < 0.001 | - 0.14 | 0.1 |
| **B10.D2 Mouse DRG SNI vs Sham** | | | | | | |
| ENSMUSG00000087301 | NA | Scn9a | -1.36 | < 0.001 | - 0.02 | 0.9 |
| **Human IPSC-derived neurons vs IPSC** | | | | | | |
| ENSG00000269890 | NA | OBSCN | 3.12 | < 0.001 | -0.45 | 0.006 |
| ENSG00000269934 | NA | OBSCN | 3.68 | 0.004 | -0.45 | 0.006 |
| ENSG00000236107 | LOC101929680 | SCN9A | 5.54 | < 0.001 | 6.14 | < 0.001 |
| LncRNA2728 | 12:51591357-51592521(-) | SCN8A | 4.76 | < 0.001 | 1.9 | < 0.01 |
