# Supplementary material for: Comprehensive analysis of long noncoding RNA expression in dorsal root ganglion reveals cell-type specificity and dysregulation after nerve injury
Source: Pain. 2018 Oct 16;160(2):463–85. doi: 10.1097/j.pain.0000000000001416 (PMC6343954; doi:10.1097/j.pain.0000000000001416)
Supplement: SUPPLEMENTARY MATERIAL [file jop-160-463-s019.doc]

| Intergenic LncRNAs close and highly correlated with pain genes | | | | | | | | | |
| --- | --- | --- | --- | --- | --- | --- | --- | --- | --- |
| LncRNA ID | LncRNA name (coordinates) | Gene symbol | Distance | LncRNA Log2 fold change | LncRNA adj. p.value | Gene Log2 fold change | Gene adj. p.value | Correlation | Cor. Adj. p.value |
| LncRNA3468 | 19:59238371-59246400(+) | Kcnk18 | -1001 | -0.46 | < 0.001 | -0.28 | 0.008 | 0.97 | < 0.001 |
|  |  |  |  | -0.19 | 0.19 | -0.08 | 0.6 |  |  |
| LncRNA4714 | 4:132078749-132109721(-) | Oprd1 | -1005 | -0.77 | < 0.001 | -0.70 | < 0.001 | 0.96 | < 0.001 |
|  |  |  |  | -0.66 | < 0.001 | -0.8 | < 0.001 |  |  |
