# Supplementary material for: Comprehensive analysis of long noncoding RNA expression in dorsal root ganglion reveals cell-type specificity and dysregulation after nerve injury
Source: Pain. 2018 Oct 16;160(2):463–85. doi: 10.1097/j.pain.0000000000001416 (PMC6343954; doi:10.1097/j.pain.0000000000001416)
Supplement: SUPPLEMENTARY MATERIAL [file jop-160-463-s020.doc]

| LncRNA | Forward | Reverse | | RT primer |
| --- | --- | --- | --- | --- |
| **Mouse** | | |  | |
| LncRNA2754 | GTAGTGCAAGCTTTGTCGTGG | TTGCGTGCTGCATTGGTATT | | TTGCGTGCTGCATTGGTATT |
| LncRNA1528 | TTAACTCCATGGCTCTCGGC | GCCTAAGGCAGGTCACACAT | | - |
| LncRNA1779 | CCTGGTGGCCATAAGGTGAG | CAGAGCATTGGGGGCTACAA | | - |
| LncRNA1291 | TCGCAGACCTCACTACCTTC | GGAGGGGTTATGTTTCCTGGAT | | - |
| LncRNA4834 | AGGCACGATGTCTGAAGCAA | TGGGAGGAGCAGTGTTAGGA | | - |
| LncRNA4714 | ATGCACAGCCAACAAACACTC | ATCCTCTCCCCTGAACCTCAT | | - |
| LncRNA561 | ATGAATGCAGCCTGACCACT | CATTCTCAGCAGGGCCAGTA | | - |
| Housekeeping | | |  | |
| HPRT1 | GTCCTGTGGCCATCTGCCTAG | TGGGGACGCAGCAACTGACA | | TGGGGACGCAGCAACTGACA |
| **Human** | | | | |
| HAGLR | CGCCCTTTCTGACCTGCTTA | TGGCAGTCGTCTGGACATTC | | - |
| Housekeeping | | |  | |
| GADPH | AGGGCTGCTTTTAACTCTGGT | CCCCACTTGATTTTGGAGGGA | | - |
| YWHAZ | CCTGCATGAAGTCTGTAACTGAG | GACCTACGGGCTCCTACAACA | | - |
